# A Non‐G‐Quadruplex DNA Aptamer Targeting NCL for Diagnosis and Therapy in Bladder Cancer

**DOI:** 10.1002/adhm.202300791

**Published:** 2023-06-22

**Authors:** Yunyi Liu, Bei Hu, Xiaming Pei, Juan Li, Dan Qi, Yuxi Xu, Hailong Ou, Yatao Wu, Lei Xue, Jason H. Huang, Erxi Wu, Xiaoxiao Hu

**Affiliations:** ^1^ State Key Laboratory of Chemo/Biosensing and Chemometrics College of Biology Molecular Science and Biomedicine Laboratory and Aptamer Engineering Center of Hunan Province Hunan University Changsha Hunan 410082 China; ^2^ Research Institute of Hunan University in Chongqing Chongqing 401120 China; ^3^ Shenzhen Research Institute Hunan University Shenzhen Guangdong 518000 China; ^4^ Hunan Yonghe‐sun Biotechnology Co. Ltd. Changsha Hunan 410082 China; ^5^ Department of Urology Hunan Cancer Hospital and The Affiliated Cancer Hospital of Xiangya School of Medicine. Changsha Hunan 410013 China; ^6^ Department of Pathology Hunan Cancer Hospital and The Affiliated Cancer Hospital of Xiangya School of Medicine. Changsha Hunan 410013 China; ^7^ Department of Neurosurgery and Neuroscience Institute Baylor Scott & White Health Temple TX 76508 USA; ^8^ Department of Medical Education Texas A&M University School of Medicine College Station TX 77843 USA; ^9^ Department of Pharmaceutical Sciences Texas A&M University School of Pharmacy College Station TX 77843 USA; ^10^ LIVESTRONG Cancer Institutes and Department of Oncology Dell Medical School The University of Texas at Austin Austin TX 78712 USA

**Keywords:** autophagy, bladder cancer, DNA aptamer, metastasis, NCL, prognosis

## Abstract

Bladder cancer (BC) is a highly aggressive malignant tumor affecting the urinary system, characterized by metastasis and a poor prognosis that often leads to limited therapeutic success. This study aims to develop a novel DNA aptamer for the diagnosis and treatment of BC using a tissue‐based systematic evolution of ligands by an exponential enrichment (SELEX) process. By using SELEX, this work successfully generates a new aptamer named TB‐5, which demonstrates a remarkable and specific affinity for nucleolin (NCL) in BC tissues and displays marked biocompatibility both in vitro and in vivo. Additionally, this work shows that NCL is a reliable tissue‐specific biomarker in BC. Moreover, according to circular dichroism spectroscopy, TB‐5 forms a non‐G‐quadruplex structure, distinguishing it from the current NCL–targeting aptamer AS1411, and exhibits a distinct binding region on NCL compared to AS1411. Notably, this study further reveals that TB‐5 activates NCL function by promoting autophagy and suppressing the migration and invasion of BC cells, which occurs by disrupting mRNA transcription processes. These findings highlight the critical role of NCL in the pathological examination of BC and warrant more comprehensive investigations on anti‐NCL aptamers in BC imaging and treatment.

## Graphical abstract

This research highlights the development of a non‐G‐quadruplex, nucleolin‐targeting aptamer TB‐5, which inhibits the invasion, migration, and proliferation of bladder cancer cells by disrupting mRNA transcription processes. TB‐5 demonstrates potential as a diagnostic and therapeutic tool due to its high specificity and affinity for bladder cancer tissues, elucidating the significant role of nucleolin in bladder cancer pathology.

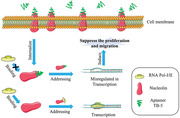

## 1 Introduction

Bladder cancer (BC) is a severe urinary system malignancy and has become the 10th most diagnosed cancer worldwide.^[^ ^1^ ^]^ Urothelial carcinomas of BC can be divided into two major subtypes, non‐muscle‐invasive bladder cancer (NMIBC) and muscle‐invasive bladder cancer (MIBC), which have completely different biological characteristics and therapeutic strategies.^[^ ^2^ ^]^ Morphologically, BC can be divided into papillary, solid, and mixed types.^[^ ^3^ ^]^ The papillary type is predominant, especially in NMIBC. Globally, the morbidity ratio of NMIBC to MIBC cases is ≈3:1; however, compared with NMIBC, most MIBC cases are higher grade and induce a higher fatality rate.^[^ ^4^ ^]^ BC has characteristics such as rapid malignant development, strong invasion, early metastasis, and high clinical stage. In the clinic, invasion and metastasis are still the key obstacles to BC therapeutics,^[^ ^5^ ^]^ and few targeted therapies are available. Due to its rapid occurrence and lack of early diagnostic means, the majority of BC patients are in the middle and late stages. Therefore, it is urgent to elucidate the pathogenesis of BC and identify new biomarkers or methods for BC diagnosis and therapy.

Aptamers are short single‐stranded DNA or RNA molecules evolved from Systematic Evolution of Ligands by Exponential enrichment (SELEX) that bind to specific molecules with high affinity. To date, different aptamers have been identified via cell‐SELEX in various cancer cells. However, cancer pathogenesis and invasion can be impacted by the microenvironment with blood vessels and matrix cells surrounding tumor cells.^[^ ^6^ ^]^ Compared to cell‐SELEX, tissue‐SELEX uses pathological tissue slides to perform in situ screening, which includes tumor sites, extracellular matrix, membrane components, and intracellular targets.^[^ ^7^, ^8^, ^9^ ^]^ Conventional tissue‐SELEX normally takes multiple repetitive rounds, which are inefficient and time‐consuming. X‐Aptamers (X‐As) are bead‐based and single‐round selection processes that can be completed rapidly without the use of any specialized instruments.^[^ ^10^ ^]^ X‐As have already been used in aptamer selections for protein targets, such as programmed cell death–1 (PD‐1)/ programmed death–ligand 1(PD‐L1).

Nucleolin (NCL) is a B–cell lymphoma 2 (BCL2)‐mRNA‐binding protein involved in cell survival, growth and proliferation. It has one of the most abundant non‐ribosomal proteins in the nucleolus and consists of three different amino acid structure regions, the N‐terminal domain, the central domain and the C‐terminal domain.^[^ ^11^ ^]^ NCL is also overexpressed in the cell membrane and cytoplasm of various cancer cells. NCL can participate in different signal transduction pathways and affect the survival, proliferation and metastasis of malignant tumors.^[^ ^12^, ^13^ ^]^ Therefore, NCL has been regarded as a potential target for cancer. For example, as one of the programmed cell death modes, autophagy is essential for maintaining cell homeostasis. NCL can inhibit cell autophagy by activating the PI3K/Akt pathway via Akt phosphorylation.^[^ ^14^ ^]^ Invasion and metastasis are important for the high mortality and poor prognosis of cancer patients. Liu et al. found that tRNA‐derived fragments can promote oligomerization of RNA‐binding proteins to form transcriptionally stable ribonucleoprotein complexes, which drive specific metabolic pathways in breast cancer progression.^[^ ^15^ ^]^ NCL is also a key regulatory factor involved in human neuroblastoma stem cell activity, revealing the potential role of NCL in anti‐cancer stem cell (CSC) therapy in neuroblastoma patients.^[^ ^16^ ^]^ Drugs targeting NCL, such as salinomycin, can effectively kill CSCs, which is important for cancer drug‐resistance therapy and prognosis.^[^ ^17^ ^]^ The biological function of NCL in promoting cellular homeostasis is also responsible for the development of its malignant features under pathological conditions.

Aptamer AS1411 has shown promise as a tumor‐targeting agent and holds potential for cancer treatment.^[^ ^18^ ^]^ However, its full efficacy in cancer treatment may be limited due to its binding to NCL through the morphological structure of the G–quadruplex (G4) chain.^[^ ^19^ ^]^ While AS1411 has demonstrated inhibitory effects on cancer in preclinical models, including breast, renal, and lung cancer, a phase II trial for renal cell carcinoma revealed minimal activity in a random patient population. This was attributed to suboptimal pharmacokinetics (rapid clearance) and low potency of AS1411, indicating challenges in its clinical application.^[^ ^20^ ^]^ Additionally, the cytotoxic mechanism of AS1411 remains unclear, and further research is needed to explore both NCL‐dependent and NCL‐independent biological effects in the future. Nevertheless, considering the crucial role of NCL in cancer development, numerous studies have investigated NCL as a key receptor for therapeutic agents and imaging probes.^[^ ^21^, ^22^, ^23^ ^]^ In this study, we employed the X‐As strategy on clinical bladder cancer tissues to generate a novel single‐stranded DNA aptamer named TB‐5. Furthermore, we investigated the target protein NCL and elucidated its recognition mechanism in BC cells. Through a competition assay between TB‐5 and AS1411, we discovered that TB‐5 could act synergistically with AS1411 by binding to different NCL binding sites, leading to a stronger anticancer effect. Compared to AS1411, TB‐5 possesses a more favorable molecular weight, a distinct binding pattern, and improved serum stability in vivo. These attributes may overcome the limitations associated with AS1411 and enhance our understanding of the biological structure of NCL. Based on our findings, TB‐5 holds potential as a novel molecular diagnostic prognostic tool and offers a new strategy based on the interaction between TB‐5 and NCL for BC therapy.

## 2 Results

### 2.1 Identification of an Aptamer Targeting BC Tissue Using the X‐As Strategy

**Figure 1:**
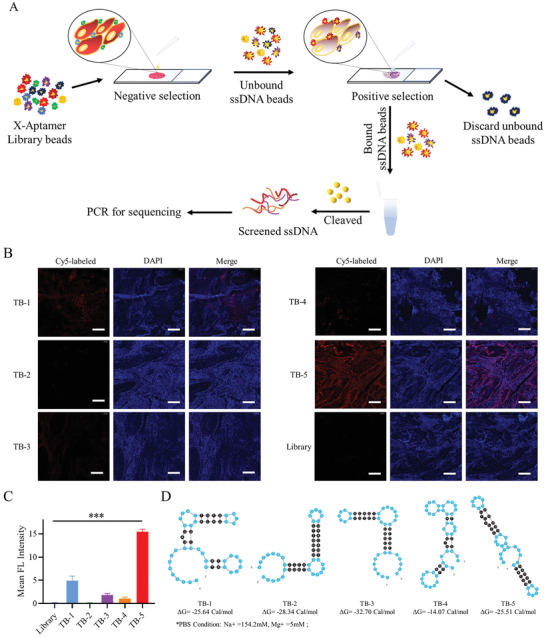
Rapid aptamer screening based on X‐As. A) Schematic illustration of the single‐round selection process against BC tissues based on the X‐As library. B) Cy5‐labeled candidate sequences or libraries were incubated with BC tissue slides. C) Statistical evaluation of fluorescence (FL) image results from B (***p < 0.005). D) Secondary structure analysis of candidate sequences was predicted by Mfold Software.

Conventionally, aptamers are evolved from a synthesized library pool. After multiple cycles of screening and PCR amplification, target‐specific aptamers can be enriched and identified by next‐generation sequencing. However, to obtain aptamers specifically, rapidly, and effectively against BC tissues, we used a novel single‐round strategy with X‐As, a modified bead‐based library pool, schematically illustrated in **Figure** **1**A. After PCR optimization of cycle number and temperature (Figure S1A,B, Supporting Information), we analyzed gel electrophoresis of different PCR samples for sequencing. Six sequences with the highest frequency were selected from different families after bioinformatic analysis (Figure S2A,B, Supporting Information). Six representative candidates (Seq1, Seq2, Seq5, Seq12, and Seq30) were chosen and synthesized for further binding ability investigation. Using a fluorescence panoramic scanner, we assessed their binding abilities to BC tissues (Figure 1B). Compared with other candidate sequences, seq30 displayed the highest binding affinity to BC tissues and was named TB‐5 (Figure 1C). The secondary structure of candidate sequences was predicted by mFold software^[^ ^24^ ^]^ (Figure 1D).

### 2.2 Clinical Tissue Array Imaging with TB‐5

**Figure 2:**
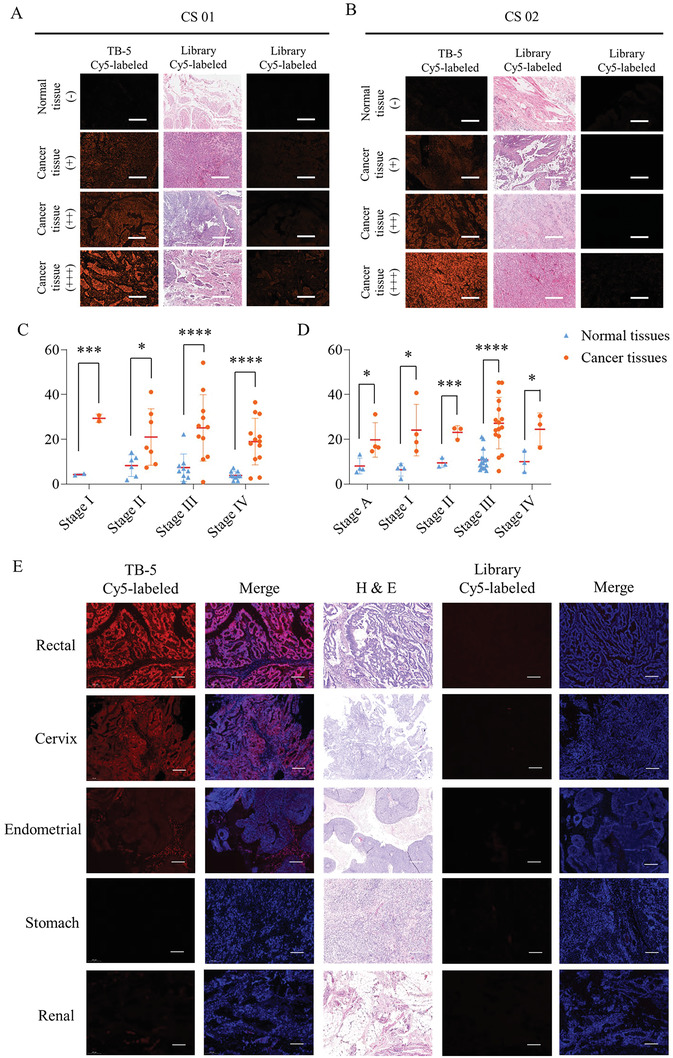
Identification of aptamer TB‐5 specifically targeting BC tissues. BC tissue arrays of A) CS01 and B) CS02 were incubated with Cy5‐labeled TB‐5 or Cy5‐labeled Library by fluorescence images with Panoramic scan (scale bar = 400 µm). Summary statistics of the relative fluorescence intensity of normal bladder and BC tissues in the C) CS01 and D) CS02 arrays, respectively. Comparison: Normal tissues versus cancer tissues: *p < 0.05, **p < 0.01, ***p < 0.005, ****p < 0.001. E) Fluorescence images of tissue sections from the various cancers stained with Cy5‐labeled TB‐5 or Cy5‐labeled Library.

To test the clinical application potential of TB‐5, we used two types of 60‐core tissue arrays (CS01, CS02), containing 63 BC cases and 57 normal bladder cases in total, with Cy5‐labeled TB‐5 or Cy5‐labeled libraries, respectively. With the fluorescence values divided into four levels, the CS01 tissue array or CS02 tissue array with Cy5‐labeled TB‐5 displayed positive fluorescence signals in 87.88% and 96.67% of bladder cancer tissues, respectively (**Figure** **2**A,B, Table S3, Supporting Information). Correspondingly, the recognition rate of the Cy5‐labeled Library to BC tissues was 42.42% in CS01 and 0% in CS02 (Table S4, Supporting Information). We also statistically analyzed the mean fluorescence intensity from adjacent normal tissues and BC tissues at different stages with four intragroup comparisons of the P value below 0.05 (Figure 2C,D, Tables S5 and S6, Supporting Information). TB‐5 can specifically recognize a range of clinical BC tissues but not normal bladder tissues, suggesting that TB‐5 may provide auxiliary clinical guidance for BC diagnosis.

We further evaluated whether it could distinguish cancer tissues from other organs, such as the rectum, stomach, endometrium, kidney, and cervix. After incubation with Cy5‐labeled TB‐5, strong fluorescence signals were observed in rectal cancer tissues and cervix cancer tissues, while mild fluorescence signals were observed in endometrium cancer tissues, and few fluorescence signals were observed in renal cancer tissues and stomach cancer tissues (Figure 2E). TB‐5 also has the potential to be a novel molecular probe for assessing various cancers.

### 2.3 Characteristics of TB‐5

**Figure 3:**
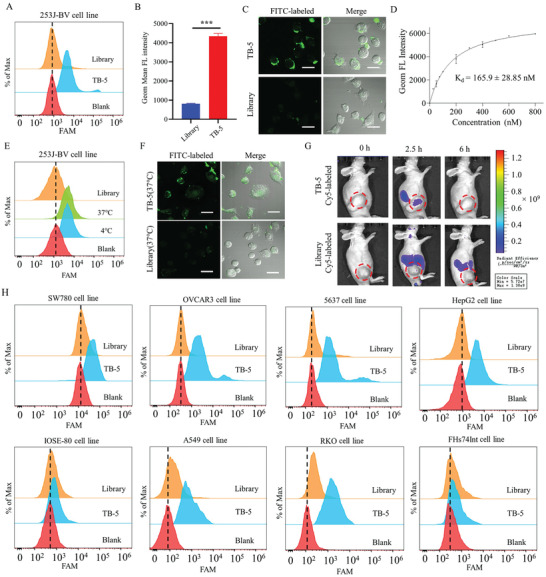
Characterization and binding ability of aptamer TB‐5. A) 253J‐BV cells were incubated with FITC‐labeled TB‐5 at 4 °C, with FITC‐labeled Library or cells used as a control. The binding ability was analyzed by flow cytometry. B) The corresponding quantification of Geom Mean FITC intensity. (FL, fluorescence). Comparison, TB‐5 versus Library: ***p < 0.005. C) FITC‐labeled TB‐5 (250 nm) binding to 253J‐BV cells was displayed by confocal microscopy, with Library used as a control (Scale bar = 40 µm). D) The dissociation constant of TB‐5 in 253J‐BV cells was analyzed by flow cytometry. 253J‐BV cells were incubated with FITC‐labeled TB‐5 at 4 or 37 °C by E) flow cytometry and F) confocal microscopy. G) BC‐bearing xenografted mice were intravenously injected with Cy5‐labeled TB‐5 (upper panel) or Cy5‐labeled Library (lower panel). Fluorescence images were taken at different time points by an in vivo imaging system. H) The binding abilities of TB‐5 to various cell lines by flow cytometry.

To obtain a robust molecular probe, we examined the characteristics of aptamer TB‐5. The recognition performance of TB‐5 with 253J‐BV cells was evaluated by flow cytometry (**Figure** **3**A). By geometric mean fluorescence (GMF) analysis and confocal microscopy imaging, FITC‐labeled TB‐5 showed strong binding signals to 253J‐BV cells (Figure 3B,C). Moreover, to verify the binding ability of TB‐5, the dissociation constant (*K*_d_) of TB‐5 to 253J‐BV cells was estimated as *Y* = *B*_max_ *X*/ (*K*_d_ + *X*). TB‐5 can bind to 253J‐BV cells with high affinity, with a *K*_d_ value of 165.9 ± 28.85 nm (Figure 3D).

Furthermore, we examined the temperature effect on TB‐5 by flow cytometry analysis. The fluorescence signals from FITC‐labeled TB‐5 started on the membrane of 253J‐BV cells equally at 37 and at 4 °C (Figure 3E). After 2 h of incubation, fluorescence signals mainly stayed at the membrane of 253J‐BV cells at 4 °C but were internalized at 37 °C, which was confirmed by confocal images (Figure 3F). Furthermore, we pretreated 253J‐BV cells with M‐*β*‐CD, amiloride or chlorpromazine to block caveolae, micropinocytosis or clathrin‐mediated pathway function, respectively, and then incubated them with FITC‐labeled TB‐5 but detected no significant change in fluorescence signals by flow cytometry (Figure S3A–C, Supporting Information). However, after pretreatment with cytochalasin D to inhibit actin polymerization, 253J‐BV cells were then incubated with FITC‐labeled TB‐5 and showed a significant decrease in fluorescence signals, suggesting that actin may be involved in the internalization of TB‐5 (Figure S3D, Supporting Information).

The stability of TB‐5 in blood circulation is essential for its application in vivo. We evaluated the nuclease resistance of TB‐5 by incubation in 10% FBS medium at 37 °C at different time points (Figure S3E, Supporting Information). The half‐life of TB‐5 was calculated to be 12.93 ± 3.845 h after agarose gel electrophoresis (Figure S3F, Supporting Information). Then, to explore the in vivo recognition of TB‐5 with BC, we performed tumor imaging by tail vein injection of Cy5‐labeled TB‐5 or Cy5‐labeled Library into xenografted mice. Fluorescence signals were captured accordingly at 0, 2.5, and 6 h post‐injection. Fluorescence signals reached a peak in the tumor site at 2.5 h post‐injection and then disappeared at 6 h post‐injection, indicating that TB‐5 can accumulate at the tumor site and further be removed via metabolism (Figure 3G, upper). By contrast, fluorescence signals were not observed at the tumor site after the injection of Cy5‐labeled Library (Figure 3G, lower), suggesting that Library cannot target tumor sites. Then, the biological distribution of Cy5‐labeled TB‐5 or Cy5‐labeled Library was observed 2 h after injection in xenograft mice. As shown in Figure S4, Supporting Information, compared with the Library, after injection of Cy5‐labeled TB‐5, fluorescence signals in mice accumulated in tumors, indicating that TB‐5 could accurately target tumor sites. In addition, mice injected with Cy5‐labeled TB‐5 or Library had a strong fluorescent signal in the kidneys because it could be rapidly metabolized and eliminated by the kidneys.

To validate the binding specificity of TB‐5 against different tumor cells, we incubated various cell lines with FITC‐labeled TB‐5 separately and then measured fluorescence signals in each cell line by flow cytometry. As shown in Figure 3H, TB‐5 not only had high affinity for other BC cell lines, such as 5637 and SW780, but also for the ovarian cancer cell line Ovcar3, breast cancer cell line MCF‐7, colon cancer cell line RKO, lung cancer cell line A549 and liver cancer cell line HepG2. However, it had low binding ability to the small intestinal epithelial cell line FHs74Int. These results indicate that the target of TB‐5 may be commonly expressed in these cancer cell lines.

### 2.4 TB‐5 Binding Target Identification

**Figure 4:**
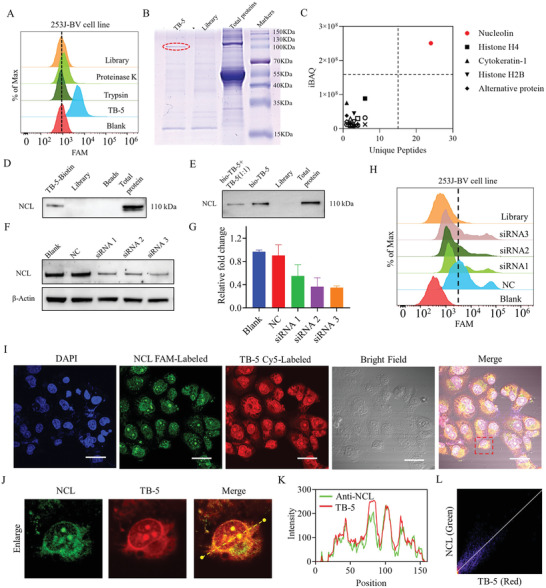
Identification of aptamer TB‐5 binding target on BC. A) Identification of target type by trypsin or proteinase K treatment. B) SDS–PAGE was performed with Coomassie blue staining to analyze the protein samples. In lane "TB–5," the protein captured from a sample treated with biotin–labeled TB–5 was loaded. The lane labeled "Library" contains the protein captured from a sample treated with biotin–labeled Library. Finally, in the "Total protein" lane, the membrane proteins extracted from 253J–BV cells were loaded for analysis. C) Mass spectrometry (MS) results of the 20 top‐ranked proteins. The abscissa is the unique peptides (%) of proteins, and the ordinate is the result of the iBAQ score. D) Western blot analysis of NCL in samples with Library or TB‐5 treatment. E) Western blotting was used for analysis after adding unlabeled TB‐5 as a competitor (bio‐TB‐5 + TB‐5). F) Knockdown levels of NCL in 253J‐BV cells. The NCL expression level (upper), NC, negative control, with *β*‐Actin as the internal control (lower). G) The corresponding quantification of E. H) Binding of TB‐5 to NCL‐downregulated cells with or without treatment was analyzed by flow cytometry. I) The subcellular localization of TB‐5 and NCL in fixed 253J‐BV cells was visualized by Cy5‐labeled TB‐5 and FITC‐labeled anti‐NCL antibody (Scale bar = 40 µm). J) Enlarged view of intracellular fluorescence signals in colocalization in (I). K) Fluorescence intensity profile of yellow arrow (see J) regions in 253J‐BV cells. L) A 2D intensity histogram of the subcellular localization is displayed.

The target of TB‐5 may exist in the cell membrane as well as the cytoplasm. Proteinase K or trypsin was used to determine whether the target type was protein. TB‐5 completely lost its binding ability to 253J‐BV cells after treatment with trypsin or proteinase K for 2 min (**Figure** **4**A), indicating that the target type of TB‐5 may be a membrane protein.

To investigate the target protein of TB‐5, we used quantitative proteomics analysis as previously reported.^[^ ^25^ ^]^ We first confirmed that biotin and FITC‐labeled TB‐5 still maintained their binding ability to 253J‐BV cells by flow cytometry assay (Figure S3G, Supporting Information). We then incubated total membrane proteins in 253J‐BV cells with biotin‐labeled TB‐5 or biotin‐labeled Library separately, which enabled potential target proteins to be captured by streptavidin‐coated sepharose beads. As shown in Figure 4B, after a differential band at 100 kDa was cut, trypsin‐digested and identified by liquid chromatography with tandem mass spectrometry (LC‐MS/MS) analysis, we collected representative peptides of related proteins (Figure S5, Supporting Information) and a list of protein hits (Table S7, Supporting Information). By the ratio of proteins captured by biotin‐labeled TB‐5 to biotin‐labeled Library, NCL may be the target of TB‐5 with a relatively higher intensity–based absolute protein quantification (iBAQ) score and more unique peptides (Figure 4C). We further confirmed that the markedly different bands captured from total protein in 253J‐BV cells by TB‐5 and by Library were specifically recognized by the anti‐NCL antibody (Figure 4D). Interestingly, this different band was significantly weakened in the lane where unlabeled competitive TB‐5 was added (Figure 4E).

Furthermore, we adopted a loss‐of‐function analysis with three NCL‐specific small interfering RNAs (siRNAs). As shown in Figure 4F,G, these siRNAs displayed a significant NCL‐knockdown effect compared with the control siRNAs. With downregulation of the cellular NCL level with siRNAs, the binding ability of TB‐5 to 253J‐BV cells decreased, as shown by flow cytometry (Figure 4H). To examine whether the colocation of TB‐5 and NCL exists, 253J‐BV cells were fixed and incubated with anti‐NCL antibody conjugated to fluorescein isothiocyanate (FITC) and Cy5‐labeled TB‐5, respectively, where intensity profiles with their overlapping were amplified in Figure 4I,J), and their Pearson's coefficient was 0.83 (Figure 4K,L). Altogether, we demonstrated that NCL may be the binding target of TB‐5 on 253J‐BV cells.

### 2.5 Binding Mechanism Exploration between TB‐5 and NCL

**Figure 5:**
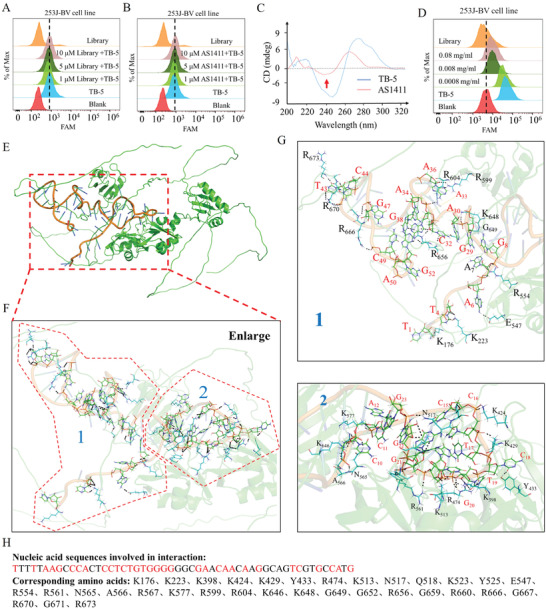
Exploring the binding mechanism between TB‐5 and NCL. 253J‐BV cells were pre‐incubated with the indicated concentration of A) AS1411 or B) Library before being incubated with FAM‐labeled TB‐5 (250 nm). Competition binding was analyzed by flow cytometry. C) CD spectra of AS1411and TB‐5. The red arrow indicates the location of the characteristic peak of AS1411. D) Competition binding between anti‐NCL antibody and TB‐5 was analyzed by flow cytometry. E) Simulation of binding modes between NCL and TB‐5. F) Enlarged view of the binding area. G) Simulated binding sites between NCL and TB‐5. Two representative binding sites were enlarged and marked as 1 and 2. H) Nucleic acid sequences and corresponding amino acids involved in the docking model.

Guanine‐rich oligonucleotides, G‐quadruplexes such as AS1411, are natural ligands of NCL, which may be due to their non‐sequence‐specific effects. To determine whether TB‐5 and AS1411 have the same binding sites by flow cytometry, we then used a competition assay in 253J‐BV cells with increasing amounts of unlabeled AS1411 or Library and a set amount of FAM‐labeled TB‐5, but the binding ability of TB‐5 was not affected (**Figure** **5**A,B).

To determine whether TB‐5 is a G‐quadruplex, we performed circular dichroism (CD) spectral analysis. As shown in Figure 5C, the CD spectrum features of TB‐5 with a positive peak at 275 nm and a negative peak at 245 nm were different from those of AS1411, suggesting that the spatial structure of TB‐5 is not a G‐quadruplex. After incubation with an increasing concentration of anti‐NCL antibody, FITC‐labeled TB‐5 gradually lost its binding to 253J‐BV cells, as shown by flow cytometry (Figure 5D), suggesting that TB‐5 has the same binding sites as the anti‐NCL antibody.

To further investigate the binding sites and interacting modes between TB‐5 and NCL, we carried out molecular docking by AutoDock software. A total of 100 protein‐aptamer interaction phases were output, and the maximum possible structural conformation (the most enriched clustering and highest score) was selected for final simulation (Figure 5E,F). The interaction between TB‐5 and NCL was divided into two regions in NCL with potential key binding sites of TB‐5 (Figure 5G,H), which could form a strong salt bridge‐hydrogen bond. Furthermore, we found that the amino acid residues of NCL proteins involved in the interaction region were mainly in the RNA‐recognition motifs (RRMs) of NCL (Figure S7, Supporting Information). NCL was reported to interact with the untranslated region of numerous mRNAs, enhancing their stability by binding with RRMs.^[^ ^26^, ^27^ ^]^ Therefore, these results suggest that TB‐5 may have a therapeutic effect by antagonizing RRMs of NCL.

### 2.6 TB‐5 Binding of NCL on the Cell Surface

**Figure 6:**
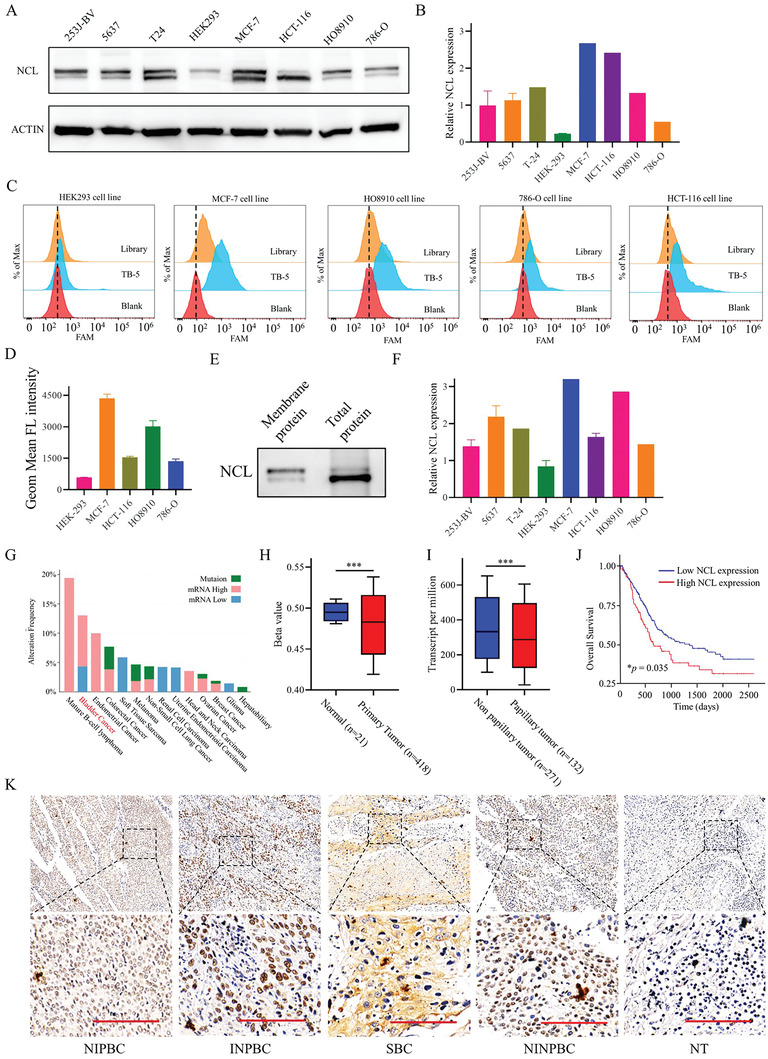
The high expression of NCL is related to BC. A) Western blot analysis of NCL expression levels in various cell lines. B) Quantified data of Western blotting results. C) The binding ability of TB‐5 to corresponding cell lines was analyzed by flow cytometry. D) Quantified data of flow cytometry results. E) Western blot analysis of NCL expression in the membrane protein and total protein extract of T24 cells. F) Quantified data of Western blotting results in the membrane protein extract of various cell lines. G) NCL gene expression frequency in pan‐cancers from the cBioPortal database. H) NCL expression level in BC (418 cases) and normal (21 cases) from the TCGA database. At least 200 cases were included in each type of cancer. Comparison, primary tumor versus normal: ***p < 0.005. I) Promoter methylation of NCL in each type of bladder cancer (Data from TCGA database). Comparison, papillary tumor versus non papillary tumor: ***p < 0.005. J) Kaplan–Meier curve in BC patients with different levels of NCL from the TCGA database. K) Representative IHC staining images of NCL levels in NIPBC, INPBC, SBC, NINPBC, and NT tissues. Scale bar = 100 µm.

To determine whether the binding affinity of TB‐5 is associated with the expression levels of NCL, we extracted total protein from four different cell lines and analyzed the expression level of NCL by Western blotting. NCL exhibited high expression in the human breast cancer cell line MCF‐7, human colon cancer cell line HCT‐116 and human ovarian cancer cell line HO‐8910 but not in the human renal cancer cell line 786‐O and human embryonic kidney cell line HEK293 (**Figure** **6**A,B).

To validate the binding affinity of TB‐5 corresponding to the NCL level, we incubated the above cell lines with FITC‐labeled TB‐5 and then individually measured their fluorescence signals by flow cytometry. TB‐5 displayed strong binding to MCF‐7 cells but not to HEK293 cells or 786‐O cells (Figure 6C,D). However, TB‐5 showed weak binding to HCT‐116, in which the expression of NCL is relatively high but in two isoforms. Considering that different functions may be related to its cellular location, we hypothesized that one isoform may be on the membrane and the other may be in the cytoplasm. NCL expression in membrane protein is the upper band from total protein in HCT‐116 cells, as shown in Figure 6E. We checked NCL expression in membrane proteins from various cell lines (Figure 6F), which was consistent with the binding affinity of TB‐5.

Moreover, to determine the binding affinity of TB‐5 on the NCL protein, we purified recombinant NCL protein (Figure S8D, Supporting Information) and employed electrophoretic mobility shift assay (EMSA) for further examination. The results indicated that TB‐5 formed an NCL/TB‐5 complex after incubation with purified NCL protein, as evidenced by the newly appeared band (Figure S8E, Supporting Information). It was clear that the gray value of the band increased with increasing NCL concentration in the SDS‐PAGE. A linear relationship between the gray value and the concentration of NCL was obtained in the range of 20 µg mL^−1^ to 12 000 mg mL^−1^ with a correlation coefficient of 0.8743. The linear regression equation was *Y* = 13.15 × Lg(*X*) − 11.62 [*X* = C_NCL_, µg mL^−1^], indicating that TB‐5 could directly bind to NCL with high affinity (Figure S8F, Supporting Information).

### 2.7 NCL Expression with BC Malignancy

To assess the role of NCL in the malignancy of BC, we evaluated NCL mRNA expression, mutation and contribution to overall BC survival by open‐access databases. Compared with other cancer tissues, the mRNA expression of NCL in BC tissue samples was relatively high (Figure 6G). Generally, the methylation level of genes in the nucleus will change dynamically with the development of cancer. Abnormal changes in DNA methylation are closely related to the occurrence of cancer. Exploring the methylation level of BC is essential for early cancer screening. Therefore, the methylation level of NCL in nonpapillary BC was higher than that in papillary BC (Figure 6I). The methylation level of NCL in bladder normal tissue was higher than that in primary BC tissue samples (Figure 6H). In addition, the higher level of NCL in BC patients generally led to a worse overall survival (Figure 6J).

To clarify the clinical significance of NCL in BC, we evaluated NCL expression with a BC tissue array slide, including non‐invasive papillary bladder carcinoma (NIPBC), invasive non‐papillary bladder carcinoma (INPBC), squamous bladder cancer (SBC), non‐invasive non‐papillary bladder carcinoma (NINPBC) and normal tissue (NT), from BC patients by immunohistochemical (IHC) analysis. Compared with NT, NCL expression was up‐regulated in NIPBC, INPBC, SBC, and NINPBC (Figure 6K), suggesting that NCL expression was correlated with the malignancy of BC.

### 2.8 Autophagy Promotion by TB‐5 in 253J‐BV Cells

**Figure 7:**
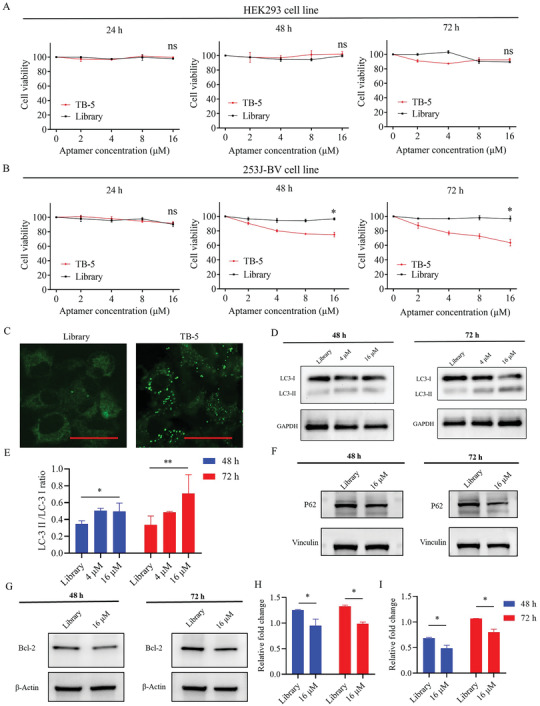
Aptamer TB‐5 affected 253J‐BV cells of BC through mitophagy. The in vitro toxicity of TB‐5 in A) HEK293 and B) 253J‐BV cells with low or high NCL expression was investigated by CCK‐8 assay. A) HEK293 and B) 253J‐BV cells were incubated with the indicated 16 µm concentrations of TB‐5 or Library. After adding CCK‐8, the cytotoxicity of 24, 48, and 72 h treatment was detected by microplate reader (Comparison, TB‐5 vs Library: *p < 0.05 ns, not significant). C) LC3‐II staining (green) exhibited punctuate dots in TB‐5‐treated 253J‐BV cells compared with Library for 72 h (Scale bar = 40 µm). D) Western blotting analysis of LC3B‐I/II and E) the corresponding quantification. F) Western blotting analysis of P62 and G) Bcl‐2. H,I) The corresponding quantification. (Comparison, 16 µm vs Library: **p < 0.01; *p < 0.05; ns, not significant).

Considering that NCL plays a key role in cell proliferation, we investigated the biological function of TB‐5 by cytotoxicity experiments. Then, 16 µm TB‐5 and Library were added to 253J‐BV cells and incubated for 24, 48, and 72 h at 37 °C. At 48 h, the viability of 253J‐BV cells treated with TB‐5 (71.32%) was significantly lower than that of cells treated with Library (98.12%). At 72 h, the viability of 253J‐BV cells with TB‐5 treatment decreased by ≈60% compared with Library treatment. The viability of 253J‐BV cells was 59.14% or 93.26% after treatment with TB‐5 and Library, respectively, suggesting that TB‐5 but not Library has a cytotoxic effect on 253J‐BV cells (**Figure** **7**A). Similar to Library, TB‐5 also displayed no significant cytotoxic effect in HEK293 cells at 24, 48, and 72 h. Collectively, TB‐5 has specific cytotoxicity to NCL high‐expressing cells.

The effect of TB‐5 on autophagy in 253J‐BV cells was also examined. 253J‐BV cells were placed in glass‐bottom chamber slides, treated with TB‐5 and Library, and transfected with baculovirus encoding LC3‐EGFP to label autophagosomes (Figure 7C). The number of mitophagosomes was increased, and the pathological changes in mitochondria were alleviated with TB‐5 treatment compared with Library treatment, as shown in TEM images (Figure S9B, Supporting Information). The expression level of LC3B‐II with TB‐5 treatment was higher than that in 253J‐BV cells with Library treatment after 48 or 72 h (Figure 7D). The expression level of P62 (SQSTM1) decreased with TB‐5 treatment compared with Library treatment in 253J‐BV cells for 48 and 72 h, respectively (Figure 7F).

### 2.9 The Migration and Invasion Suppression of TB‐5 in 253J‐BV Cells

**Figure 8:**
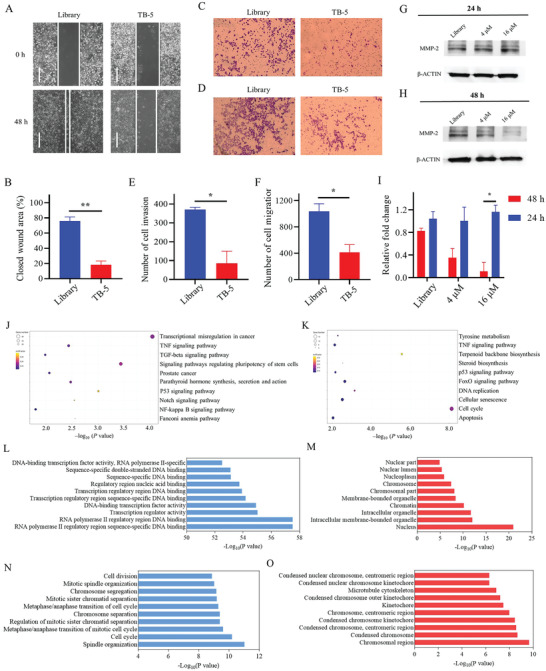
The aptamer TB‐5 inhibits BC cell migration and invasion in vitro. A) The effects of aptamer TB‐5 on the cell migration of 253J‐BV cells detected by wound healing assay. 253J‐BV cells were treated with 16 µm Library or 16 µm TB‐5 for 48 h. B) The corresponding statistical analysis of A. Comparison, TB‐5 versus Library: **p < 0.01. The effects of TB‐5 on C) invasion and D) migration in 253J‐BV cells by Transwell assay. E,F) The corresponding statistical analysis of G) and H). Comparison, TB‐5 versus Library: *p < 0.05. The effects of TB‐5 on the MMP2 signaling pathway in 253J‐BV cells were detected by Western blotting. 253J‐BV cells were treated with 16 µm Library or 16 µm TB‐5 for G) 24 h and H) 48 h. I) The corresponding statistical analysis of G and H (Comparison, 48 h versus 24 h: *p < 0.05). mRNA expression analysis of 253J‐BV cells treated with TB‐5 and AS1411. KEGG analysis in J) TB‐5 versus Library and K) AS1411 versus Library. GO set enrichment analysis of L,M) BP and CC comparing TB‐5 with N,O) Library or AS411 with Library.

Metastasis usually leads to a dismal prognosis for BC therapeutics. The 253J‐BV cell line is a bladder transitional carcinoma cell line normally used in cell migration and invasion studies. Compared with Library treatment, the migration of 253J‐BV cells was significantly inhibited with TB‐5 treatment after 48 h. (**Figure** **8**A,B). The effect of TB‐5 on cell invasion was further measured by transwell assay. The number of 253J‐BV cells crossing the transwell chamber was significantly less with TB‐5 than with Library treatment for 48 h, suggesting that TB‐5 may inhibit cell invasion (Figure 8D). TB‐5 enhanced cell invasion via matrix metallopeptidase 2 (MMP‐2), as shown in the Western blotting images (Figure 8G,H).

### 2.10 Different Signaling Pathways Regulated by AS1411 and TB‐5 in BC Cells

To identify the signaling pathways, 253J‐BV cells treated with AS1411, and TB‐5 were subjected to mRNA sequencing. Kyoto Encyclopedia of Genes and Genomes (KEGG) pathway analysis analyzed differentially expressed genes (DEGs) between TB‐5 and Library treatment separately, revealing that the genes regulated by TB‐5 were significantly enriched in pathways including transcriptional misregulation in cancer, tumor necrosis factor‐alpha (TNF) signaling, Notch signaling, NF‐kappa B signaling and transforming growth factor‐beta (TGF‐beta) signaling (Figure 8J). In contrast, the analysis of DEGs between AS1411 and Library treatment revealed that the genes regulated by AS1411 were significantly enriched in pathways including TNF signaling, apoptosis, p53 signaling, DNA replication, and the cell cycle (Figure 8K). The DEGs in detail by TB‐5 or AS1411 treatment are shown in Figure S10, Supporting Information. TB‐5 primarily participated in gene transcription regulation, while AS1411 was involved in cell apoptosis by inhibiting the cell cycle and inflammation.

Gene Ontology (GO) analysis of the biological process (BP) and cellular component (CC) ontologies also revealed that TB‐5 regulated transcriptional processes, which exist in the nucleus and cytoplasm of 253J‐BV cells (Figure 8L,M), while AS1411 regulated the cell cycle, cell division and nuclear chromosome‐related proteins, which are in the nucleus (Figure 8N,O). The GO enrichment maps of DEGs between TB‐5 and Library indicated that TB‐5 can suppress the mitigation and promote the autophagy of 253J‐BV cells through the regulation of cell transcription, while AS1411 blocks the cell cycle via the regulation of chromosome function (Figure S11, Supporting Information).

## 3 Discussion

By using a single‐round X‐A procedure, we identified a new aptamer, TB‐5, that can distinguish BC tissues from normal bladder tissues. Additionally, TB‐5 can specifically recognize BC or other cancer cell lines with high affinity and accumulate at tumor sites in xenograft mouse models, which demonstrated that it may be used in clinical targeted therapy. With high adaptability to a variety of targets and low immunogenicity, aptamers have been studied in biomarker discovery and antitumor drug delivery. We further identified the binding protein of TB‐5 as NCL, which is elevated in BC patients with poor prognosis. In contrast to another NCL‐targeting aptamer, AS1411, TB‐5 can not only promote cell autophagy but also suppress cell migration and invasion in BC, suggesting that TB‐5 can be a potential molecular probe or drug for clinical BC diagnosis and treatment.

Our findings have broadened the landscape of DNA aptamers for anticancer applications. Conventionally, an aptamer interacts with the surface structure of a target protein by folding into a unique secondary or tertiary structure, while it is easily dissociated from the protein even by small differences in surface structure. The unique properties of aptamers enable their great potential for exploring allosteric modulators of protein functions, such as ligand‐induced conformational changes.^[^ ^28^, ^29^ ^]^ In 1998, Bates et al. identified the target of the G‐rich oligonucleotide AS1411 as the NCL protein.^[^ ^30^ ^]^ AS1411 exhibited antitumor effects in a variety of cancer types, including breast cancer, kidney cancer, and lung cancer. In 2003, Dr. Damian initiated the first phase I study of AS1411 in patients with a variety of advanced solid tumors with progressive metastasis.^[^ ^31^ ^]^ To date, 11 aptamers have entered the clinical trial stage, including pegaptanib [a vascular endothelial growth factor (VEGF)‐targeting RNA aptamer], AS1411 [NCL‐targeting DNA aptamer], NOX‐A12 [chemokines, CXCL12‐targeting RNA aptamer], etc., suggesting that TB‐5 may also be a potential therapeutic drug for the future translation of our bench research into clinical trials.^[^ ^32^, ^33^ ^]^

Pathological section examination is one of the main methods for cancer diagnosis with different stages.^[^ ^34^ ^]^ Tumor staging helps to uncover risk factors of clinical relevance and statistical significance for cancer patients. NCL can be expressed in various eukaryotic cell compartments and facilitate multiple cellular functions; for example, NCL is expressed at the cancer cell surface and acts as a cell‐surface receptor for many different ligands, including growth factors (i.e., basic fibroblast growth factors) and chemokines (i.e., metaphase factors).^[^ ^35^ ^]^ The expression level of NCL in BC patients can be correlated with disease progression, suggesting that NCL could be a potential valuable prognostic marker for BC tissues.

Autophagy is essential for maintaining cell homeostasis, which can be inhibited not only by the Ras‐activated PI3K/Akt/mTOR pathway^[^ ^36^, ^37^ ^]^ but also by Bcl‐2 blocking the Beclin‐1 pathway.^[^ ^38^ ^]^ NCL can activate the cellular autophagy pathway by downregulating the autophagy‐related proteins P62 or Bcl‐2.^[^ ^39^ ^]^ TB‐5 could increase the intracellular protein level of P62 or microtubule–associated protein 1A/1B–light chain 3B (LC3II), suggesting that TB‐5 can increase the mortality of tumor cells via autophagy.

Migration and invasion of malignant tumor cells can result in high mortality and poor prognosis. The interaction between C–X–C motif chemokine ligand 12 (CXCL12) and C–X–C chemokine receptor 4 (CXCR4) plays an important role in promoting tumor growth and migration.^[^ ^40^ ^]^ NCL can effectively bind to the 212 C‐terminus of CXCR4, which activates CXCR4 signaling in tumor cell growth and synergistically improves tumor invasion and metastasis.^[^ ^41^, ^42^ ^]^ The suppressive effects of TB‐5 on BC cells occur via suppression of MMP‐2 expression.

In recent years, NCL has been reported to work with gene enhancers to regulate gene transcription.^[^ ^43^, ^44^, ^45^ ^]^ In embryonic stem cells, NCL can maintain self‐renewal by inhibiting the p53‐dependent pathway.^[^ ^46^ ^]^ Through mRNA expression profile analysis, TB‐5 regulated target cells at the transcriptional level, different from blocking the cell cycle by AS1411. Importantly, the relationship between NCL function and spatial structure helps to develop NCL‐targeting treatments for cancer and other diseases. NCL contains four RRMs and the C‐terminal glycine‐ and arginine‐rich (GAR) region. RRM3 and RRM4 independently recognize target RNAs or proteins, and RNA or protein binding via RRM3 plays a minor role in the nucleolar localization of NCL.^[^ ^47^ ^]^ NCL interacted directly with ribosomal RNAs (rRNAs) via RRM1, RRM4, and GAR. Studies have shown that the deletion of transcription factors or transcription disorders can lead to inhibition of tumor proliferation, migration, or invasion.^[^ ^48^, ^49^, ^50^ ^]^ Therefore, depending on the models of molecular docking, we hypothesized that TB‐5 interferes with the binding of the RRM region of NCL to rDNA, which disrupts the regulation of the rRNA transcription process and then inhibits the proliferation and migration of cancer cells. However, it remains to be further studied how these RNA‐binding regions of NCL separately or cooperatively regulate its functions.

In conclusion, the high specificity of TB‐5 for NCL makes it an excellent prognostic tool for cancer therapeutics. NCL directly or indirectly participates in signal transduction in malignant cancers, with different mechanisms, affecting the survival, proliferation, and metastasis of cancer cells. Currently available anti‐NCL aptamers (AS1411 and other G4s) can only activate the functions of NCL in the cell cycle and apoptosis. Thus, aptamer TB‐5, which is distinguished by a novel single‐round tissue‐SELEX, promotes autophagy and inhibits the migration and invasion of BC cells by disordering the RNA transcription process of cancer cells. Meanwhile, given the low immunogenicity of TB‐5, it can serve as an alternative to antibodies to detect cancer histological sections and therapy in vivo. Furthermore, its correlation with the clinical outcomes of BC patients may also be due to its target protein NCL. TB‐5 has great potential to serve as a targeting ligand in future work, such as clinical studies. Our study lays the foundation for functionalization of the anti‐NCL aptamer TB‐5, as well as future translational research for clinical applications.

## 4 Experimental Section

### X‐As kit, ssDNA Library, Primers, and Buffers

X‐As kit (AM Biotechnologies, Houston, TX, USA) was obtained from Sangon Biotech Co. Ltd. (Shanghai, China). The kit contains the X‐As bead library, one forward primer and four reverse primers. All DNA sequences were purchased from Hippo Biological Technology Co., Ltd. (Huzhou, China). All sequence information is shown in Table S1, Supporting Information.

Washing buffer (WB) was prepared with Dulbecco's phosphate buffered saline (D‐PBS) (HangZhou GENOM Co., Ltd) supplemented with 4.5 g L^−1^ glucose and 5 mm MgCl_2_. Binding buffer (BB) was mixed with D‐PBS supplemented with 4.5 g L^−1^ glucose, 5 mm MgCl_2_, 0.1 mg mL^−1^ yeast tRNA, and 1 mg mL^−1^ BSA. Buffer A was prepared with phosphate buffered saline (PBS) supplemented with 1 mm MgCl_2_, 0.5 µL mL^−1^ Tween 20, and 2 mg mL^−1^ BSA. Buffer B was prepared with PBS supplemented with 1 mm MgCl_2_ and 0.5 µL mL^−1^ Tween 20.

### Apparatus

All flow cytometry experiments were analyzed by the Cytek DxP Athena system, USA. The confocal microscopy graphs were imaged by a Fluoview FV1200 confocal laser scanning microscope (Olympus, Japan). CD spectra were analyzed by a Bio‐Logic MOS‐500 CD spectrophotometer (Claix, France). The fluorescence images of mice in vivo were collected by an IVIS Lumina II imaging system (Caliper LifeScience, USA). The fluorescence signals of the clinical bladder tissue array were detected by a Panoramic MIDI (3DHISTECH Ltd., Hungary). All PCR‐amplified products were high‐throughput sequenced by Illumina MiSeq (Sangon Biotech Co., Ltd. Shanghai, China). DNA gel electrophoresis and Western blot results were obtained by a ChemiDoc™ XRS Imager (Bio‐Rad, USA).

### X‐As Procedure

The clinical BC tissue and adjacent tissue used in this screening procedure were all collected from the Tumor Hospital of Hunan Province. First, the collected tissue samples were buried in embedding agent. The embedded BC tissue samples were prepared on frozen tissue slices of 50 µm and washed with D‐PBS to remove the excess embedding agent.

Second, the X‐As Library was preprocessed with a similar protocol that was reported in a previous study.^[^ ^25^ ^]^ Then, adjacent tissue slides were incubated with the pretreated X‐As Library for 60 min with rotation. Unbound library beads were then collected in the EP tube (tube #1). After negative selection, the former unbound single–stranded DNA (ssDNA) Library beads (tube #1) were incubated with BC tissue for 90 min at room temperature with rotation. Afterward, BC tissues (containing positive selection beads) were collected and resuspended in 50 µL buffer B (tube #2). To cleave the ssDNA from the selected library beads, 50 µL of 1 n NaOH was added to the 50 µL resuspension in tube #2 at 65 °C for 30 min. The reaction was neutralized by adding 40 µL of 2 m Tris‐Cl. The selected ssDNA sequences in tube #2 were enriched according to the X‐As selection protocol and then amplified by PCR.

The amplified ssDNA strands in the above tubes were analyzed by next‐generation sequencing. The sequencing data were processed using Aptaligner software.^[^ ^51^ ^]^ After the analysis of homologous similarity using DNAMAN v6.0 software, six candidate sequences were chosen for further investigation.

### Fluorescence Imaging of Clinical Pathological Section Slides

Bladder tissue array slides were preheated at 60 °C for 2 h and then deparaffinized in xylene (15 min, twice). Tissue sections were then immersed in decreasing ethanol concentrations (100%, 95%, 90%, 80%, and 70%) at 5‐min intervals. The hydrated tissues were pretreated in 0.01 m citrate buffer at pH 6.0 and heated in a pressure cooker for 20 min. Afterward, tissue sections were blocked with precooled binding buffer and 20% fetal bovine serum (FBS) for 1 h at room temperature and incubated with 250 nm Cy5‐labeled Library or Cy5‐labeled TB‐5 for 1 h. The fluorescence intensity of each case was calculated and evaluated by ImageJ (Version 1.53c) as negative (−, <10.0), weak (+, 10.0–20.0), moderate (++, 20.0–30.0), or strong (+++, >30.0).

### Flow Cytometry Analysis

The binding ability of the candidate aptamers to cells was analyzed by flow cytometry. Each cell line (2 × 10^5^ cells) was incubated with 250 nm of candidate FAM‐labeled aptamers or FAM‐labeled Library in 250 µL of binding buffer at 4 °C for 1 h. These cells were then washed with WB and resuspended in 200 µL of D‐PBS.

To measure the dissociation constant (*K*_d_), 253J‐BV cells (3 × 10^5^) were incubated with different concentrations of aptamers in 250 µL of BB at 4 °C for 1 h. After incubation, each sample was washed three times with WB and then resuspended in 200 µL of D‐PBS for flow cytometry analysis. These experiments were repeated three times. Using GraphPad Prism 8.0 software, the *K*_d_ of the aptamers was calculated by the equation *B*_max_X/ (*K*_d_ + *X*) (*Y*: relative fluorescence intensity; *X*: aptamer concentration), fitting the dependence of the fluorescence intensity of the cell/aptamer complex on the aptamer concentration.

### Confocal Microscopy Imaging

Target cells were seeded at 1 × 10^5^ in a 35‐mm glass‐bottom dish (NEST Biotechnology Co. Ltd., Wuxi, China) and cultured for 24 h. After washing with cold WB, cells were incubated with 300 nm FAM‐labeled aptamer in 1 mL of BB at 4 °C for 1 h. After washing three times, cells were imaged by confocal laser scanning microscopy.

### In Vivo Fluorescence Imaging

Male BALB/c‐nude mice (5–6 weeks old) were purchased from Changsha SLAC Animal Laboratory and raised in specific pathogen‐free (SPF) conditions. All animal experiments were performed with the approval of the Laboratory Animal Center of Hunan University. A total of 5 × 10^6^ 253J‐BV cells were implanted in subcutaneous tissue of the mouse backside. After tumors reached 0.5–1.5 cm in diameter at a period of 15–20 days, these tumor‐bearing mice were anesthetized with isoflurane and injected intravenously with 100 µL of 4.5 nm Cy5‐labeled TB‐5 or Cy5‐labeled Library (three mice for each group). After fluorescence imaging analysis at 2.5 h postinjection, tumor‐bearing mice were sacrificed and dissected. Their tumor tissues and visceral organs, including the kidneys, liver, stomach, intestine, heart, testis, and prostate, were then imaged.

### Target Protein Identification by Mass Spectrometry Analysis

The binding target of TB‐5 was investigated according to a similar protocol that was previously reported.^[^ ^25^ ^]^ The plasma membrane proteins of 253J‐BV cells were extracted by the Membrane and Cytosol Protein Extraction Kit (P0033, Beyotime, China).

### Western Blot Analysis

Total cell lysates or extracted proteins were resolved by 10% SDS‐PAGE and then transferred to PVDF membranes (Merck Millipore Ltd, ISEQ00010). PVDF membranes were then separately incubated with the primary antibody at 4 °C overnight. After washing three times with Tris‐buffered saline with 0.1% Tween 20 (TBST), the membrane was incubated with the corresponding secondary antibody. The signals were detected with Western Bright ECL (K‐12045‐D50, Advansta) and imaged by ChemiDoc XRS Imager (Bio‐Rad, USA).

### RNA Interference

The sequences of NCL siRNA (siRNA1 sense: GUACUAUACUGGAGAGAAATT; siRNA2 sense: GGAAAUGGCCAAACAGAAATT; siRNA3 sense: CGGUGAAAUUGAUGGAAAUTT) were purchased from General Biotech Co. Ltd. (Chuzhou, China). 253J‐BV cells and transfected with siRNA by Lipo6000™ transfection reagent (C0526, Beyotime, China) for 24–48 h. While the cells were harvested and analyzed by flow cytometry, their protein extracts were detected by Western blotting.

### Fluorescence Analysis of the Colocalization Experiment

First, 253J‐BV cells (8 × 10^4^) were seeded into a 35‐mm optical vessel. After incubation at 37 °C for 24 h, 253J‐BV cells were washed three times with DPBS and fixed in 4% paraformaldehyde for 15 min. Then, the cells were washed twice with DPBS and treated with anti‐NCL antibody at 4 °C overnight. After washing three times with PBS with 0.1% Tween 20 (PBST), FITC‐labeled anti‐rabbit secondary antibody was added to the fixed 253J‐BV cells at room temperature for 2 h. The cells were washed twice with PBST, and the optical vessel was stained with DAPI (10 µg mL^−1^) for 10 min.

Finally, 250 nm FAM‐labeled TB‐5 was added to the optical vessel. The supernatant was removed after incubation for 1 h. Fluorescence signals were detected by Zeiss LSM510. All experiments were repeated three times.

### Pulldown Assay

Flag‐tagged NCL was overexpressed in HEK293 cells and purified by anti‐Flag affinity gel beads (P2282, Beyotime, China) according to the manufacturer's instructions. These beads were mixed overnight at 4 °C with NCL‐overexpressing HEK293 cell lysates. After removing the supernatant by low‐speed centrifugation, the gel beads were mixed with 40 µL of protein loading buffer and heated at 95 °C for 5 min to denature proteins. Then, the purified NCL was collected and examined by SDS‐PAGE.

### Electrophoretic Mobility Shift Assay

First, binding reactions were performed in binding buffer containing purified 3×Flag‐tagged NCL proteins and 250 nm FITC‐labeled TB‐5 in a final volume of 20 µL. After mixing, the reaction was incubated at 4 °C for 1 h, and then each sample was supplemented with loading buffer and loaded onto a 4% polyacrylamide gel that had been pre‐run for 10 min. Electrophoresis was performed for 1 h at 120 V and 4 °C. Complexes and free DNA were visualized by ChemiDoc XRS Imager (Bio‐Rad, USA).

### Molecular Docking

First, the protein structure of NCL was downloaded from the AlphaFold Protein Structure Database^[^ ^52^, ^53^ ^]^ (AlphaFold model ID: AF‐P19338‐F1). The 3D structure of aptamer TB‐5 with the best predicted energy was also constructed by RNA Composer software.^[^ ^54^, ^55^ ^]^ After nucleic acid bases T were changed to U, one position‐constrained molecular dynamics simulation was performed to eliminate the steric conflicts within the aptamer system.

Second, docking simulations were performed with AutoDock software.^[^ ^56^, ^57^ ^]^ The docking protocol began with rigid‐body blind docking where all heavy atoms of the protein and aptamer systems were strictly restrained. This step randomly placed the protein and aptamer roughly facing each other and obtained at least 500 docking conformations for further analysis.

Finally, precise docking site prediction was carried out by PyMOL software^[^ ^58^, ^59^ ^]^ based on the best docking conformation of the NCL‐TB‐5 complex. Binding spots with distances beyond 4 Å were manually deleted and thus simplified to final binding spots.

### Bioinformatic Analysis

NCL gene expression frequency across cancers was collected from the cBioPortal database.^[^ ^60^, ^61^ ^]^ The analysis of the expression or methylation levels of NCL in BC patients was performed using data from the The Cancer Genome Atlas (TCGA) database^[^ ^62^ ^]^ (UALCAN, http://ualcan.path.uab.edu). The filter indices were set as differential analysis between cancer and normal specimens, with individual cancer stages and sexes.

### Immunohistochemical Staining

BC tissue array slides with adjacent BC and BC tissues were purchased from Shanghai OUTDO Biotech Co., Ltd. (HBlaU050CS01). First, the tissue array was deparaffinized with xylene and rehydrated in gradient ethanol. The array slides were immersed in sodium citrate antigen retrieval solution (pH 6.0). After blocking with serum, these slides were incubated with anti‐NCL rabbit polyclonal antibody (D127319, Sangon Biotechnology Co., Ltd.) at 4 °C overnight. These slides were incubated with HRP (rabbit) secondary antibody (D110011, Sangon Biotechnology Co., Ltd.) at room temperature for 1 h. Finally, sections were developed in freshly prepared 3,3′‐diaminobenzidine (DAB) chromogenic reagent until the nucleus showed a brownish yellow under microscopy, followed by counterstaining the cytoplasm to be blue with hematoxylin staining solution.

### Cytotoxicity Assay

Cells were seeded in 96‐well plates at 1 × 10^5^ cells per well. The cells were added to Library or TB‐5 of gradient concentration and cultured at 37 °C for 24, 48, and 72 h, respectively. After supernatant removal, these wells were washed with PBS, and CCK‐8 solution was then added. After incubation at 37 °C for 1 h, the plates were then detected by a Synergy 2.0 microplate reader (BioTek, USA). All experiments were repeated three times.

### Transmission Electron Microscopy

253J‐BV cells were harvested in 1.5 mL tubes (Guangzhou Jet Bio‐Filtration Co.), prefixed in 2% glutaraldehyde, and fixed in 1% osmium tetroxide. Next, samples were dehydrated in ethanol with 3% uranyl acetate and embedded in epoxy resin and propylene oxide overnight to polymerize. After slicing into 70 nm thick sections and staining with lead citrate, the sections were detected by transmission electron microscopy (H‐7800, Hitachi). Two blinded pathologists quantified each section independently.

### Wound Healing Assay

253J‐BV cells (5 × 10^6^) were seeded in a 6‐well plate overnight for attachment and cultured in DMEM with 1% FBS (NEWZERUM Ltd.) for 24 h. The confluent cell monolayer was then scratched uniformly with a scratching device (BioTek) after cell confluence reached 80%–90% and washed three times to remove floating cells and cell debris. Wound healing was imaged by an inverted microscope. The area of the wound gap was measured and recorded by ImageJ software.

### Transwell Assay

2 × 10^4^ cells were suspended in 200 µL complete cell culture medium (HangZhou GENOM Co., Ltd) and seeded in the upper chamber (8 µm pore size, Corning) of a 24‐well plate overnight. The next day, the medium in the upper chamber was replaced by fresh cell medium without FBS, and the lower chamber was filled with 500 µL medium containing 20% FBS to induce cell migration. The chamber was incubated at 37 °C for 3 days. Subsequently, the cells were washed with PBS three times and then fixed with 4% paraformaldehyde and 1% crystal violet for 10 min at room temperature. After washing with water, non‐migrated cells were wiped with cotton swabs, images were obtained using an inverted microscope (IX‐73, Olympus), and the number of migrated cells was counted by ImageJ.

### RNA Extraction, Library Construction, and Illumina Sequencing

253J‐BV cells (3 × 10^6^) were seeded in a 6‐well plate (Guangzhou Jet Bio‐Filtration Co.) overnight for attachment and cultured in DMEM with 10% FBS for 24 h. Then, the 6‐well plate was treated with TB‐5, AS1411, and Library for 72 h. RNA extraction and mRNA sequencing were executed by APTBIO Co., Ltd, China.

### Statistics

The statistical analysis was implemented by GraphPad Prism 8.0. Statistical significance was determined via *t*‐tests; *p < 0.05 was considered significant.

## Conflict of Interest

The authors declare no conflict of interest.

## Supporting information

Supporting Information

## Data Availability

The data that support the findings of this study are available from the corresponding authors upon reasonable request.
