# Supplementary material for: A Non‐G‐Quadruplex DNA Aptamer Targeting NCL for Diagnosis and Therapy in Bladder Cancer
Source: Adv Healthc Mater. 2023 Jun 22;12(20):2300791. doi: 10.1002/adhm.202300791 (PMC11469069; doi:10.1002/adhm.202300791)
Supplement: Supplementary file 1 — Supporting Information [file ADHM-12-2300791-s001.pdf]

ADVANCED HEALTHCARE MATERIALS

Supporting Information

for *Adv. Healthcare Mater.*, DOI 10.1002/adhm.202300791

A Non-G-Quadruplex DNA Aptamer Targeting NCL for Diagnosis and Therapy in Bladder Cancer

Yunyi Liu, Bei Hu, Xiaming Pei, Juan Li, Dan Qi, Yuxi Xu, Hailong Ou, Yatao Wu, Lei Xue, Jason H. Huang, Erxi Wu and Xiaoxiao Hu**

A non-G-quadruplex DNA aptamer targeting NCL for diagnosis and therapy in bladder cancer

Yunyi Liu[1#], Bei Hu[1#], Xiaming Pei[5], Juan Li[1], Dan Qi[7], Yuxi Xu[1], Hailong Ou[1], Yatao Wu[1], Lei Xue[6], Jason H. Huang[7,8], Erxi Wu[7,8,9,10*], Xiaoxiao Hu[1,2,3,4*]

[1] *State Key Laboratory of Chemo/Biosensing and Chemometrics, College of Biology, Molecular Science and Biomedicine Laboratory and Aptamer Engineering Center of Hunan Province, Hunan University, Changsha, Hunan 410082, China*

[2] *Research Institute of Hunan University in Chongqing, Chongqing, 401120, China.*

[3] *Shenzhen Research Institute, Hunan University, Shenzhen, Guangdong, 518000, China*

[4] *Hunan Yonghe-sun Biotechnology Co., Ltd. Changsha，Hunan 410082, China*

[5] *Department of Urology, Hunan Cancer Hospital and The Affiliated Cancer Hospital of Xiangya School of Medicine. Changsha, Hunan 410013, China*

[6] *Department of Pathology, Hunan Cancer Hospital and The Affiliated Cancer Hospital of Xiangya School of Medicine. Changsha, Hunan 410013, China*

[7] *Department of Neurosurgery and Neuroscience Institute, Baylor Scott & White Health, Temple, TX, 76508, USA*

[8] *Department of Medical Education, Texas A&M University School of Medicine, College Station, TX 77843, USA*

[9] *Department of Pharmaceutical Sciences, Texas A&M University School of Pharmacy, College Station, TX 77843, USA*

[10] *LIVESTRONG Cancer Institutes and Department of Oncology, Dell Medical School, The University of Texas at Austin, Austin, TX 78712, USA*

[#] *Contributed equally to this work.*

[*] To whom correspondence should be addressed. E-mail: erxi.wu@bswhealth.org (Erxi Wu), xxhu@hnu.edu.cn (Xiaoxiao Hu).

Table S1. DNA sequences used in this work

Name	Sequence (5' → 3')	Length (nt)
Library	TTTTTAACACG -30N- GTGGGCCCATG	52
Primer-F	TTTTTAAGCCC	11
Primer-R	GTCGTGCCATG	11
TB-1	TTTTTAAGCCCACATCGCTGTGCACAGCACAGTGCG AGCATGTCGTGCCATG	52
TB-2	TTTTTAAGCCCACTGCGGTTCGACTCACGAACAGTG ACAACGTCGTGCCATG	52
TB-3	TTTTTAAGCCCACGAGACTGTGCCGGACACAGTCCG TCAGCGTCGTGCCATG	52
TB-4	TTTTTAAGCCCACAAAACTGTGGACACCGAACCAAT GCACTGTCGTGCCATG	52
TB-5	TTTTTAAGCCCACTCCTCTGTGGGGGGCGAACAACA AGGCAGTCGTGCCATG	52

Table S2. Cell lines and culture conditions used in this work

Histologic Cell Type	Cell Lines
Human bladder carcinoma cell line	5637*
Human colon carcinoma cell line	RKO*
Human ovarian adenocarcinoma cell line	OVCAR3*
Human small intestine epithelial cell line	FHs74Int*
Human ovarian epithelial cell line	IOSE-80*
Human renal carcinoma cell line	786-O*
Human breast carcinoma cell line	MCF-7#
human embryonic kidney epithelial cell line	HEK293#
Human bladder transitional carcinoma cell line	T24#
Human bladder carcinoma cell line	253J-BV#
Human mammary epithelial cell line	MCF-10A※
Human hepatoma cell line	HepG2#
Human bladder transitional carcinoma cell line	SW780#
Human colon carcinoma cell line	HCT-116#

- All the cells were cultivated in ambient 5% CO_2 at 37 °C.
- *The cells were cultivated in RPMI 1640 with 10% FBS.

  #The cells were cultivated in DMEM with 10% FBS.

  ※The cells were cultivated in MCF-10A cell culture medium.

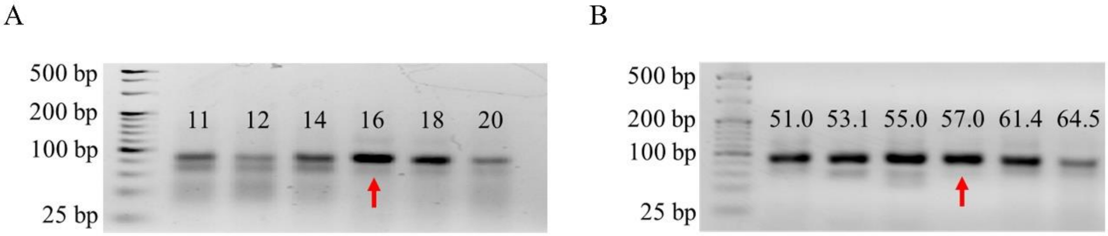

Figure S1. Condition optimization for PCR. 12% PAGE gel electrophoresis analysis of PCR products with (A) different cycle numbers and (B) different temperatures. The red arrows indicate the best condition.

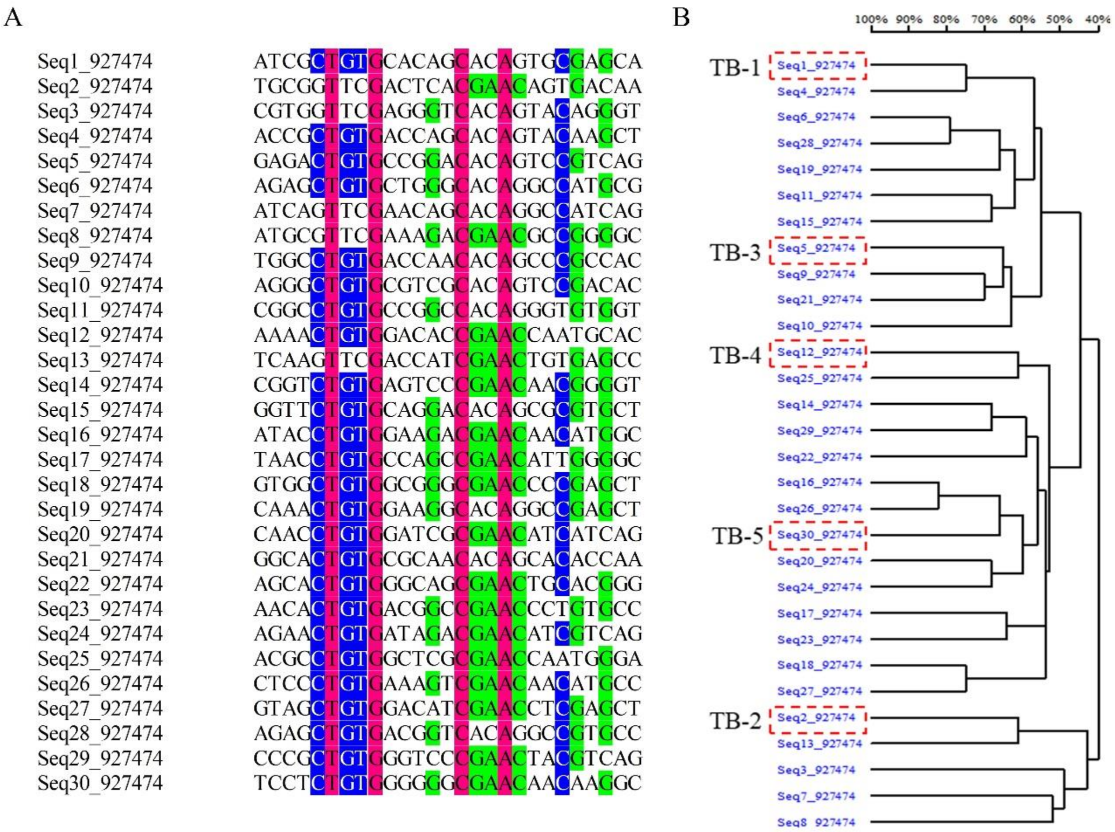

Figure S2. Homology analysis of sequences with the top 30 repeats in the sequencing results. (A) DNA primary sequence alignment analysis. (B) The evolutionary tree of DNA sequences, with candidate sequences marked in the red box.

Table S3. Summary tissue imaging results with TB-5.

TB-5	CS01	CS02
	Positive	Positive
Cancer tissue	87.88%	96.67%
Normal tissue	14.81%	33.33%

Table S4. Summary tissue imaging results with Library.

Library	CS01	CS02
	Positive	Positive
Cancer tissue	42.42%	0%
Normal tissue	3.84%	6.67%

Table S5. Assessment of the clinical tissue array CS01 stained with Cy5-labeled TB-5.

AJCC Staging System (7th edition)	T1	T2	T3	T4	Normal
-	0	2	1	1	23
+	0	2	3	6	3
++	1	1	4	2	1
+++	1	2	3	3	0

Table S6. Assessment of the clinical tissue array CS02 stained with Cy5-labeled TB-5.

AJCC Staging System (7th edition)	TA	T1	T2	T3	T4	Normal
-	0	0	0	1	0	20
+	3	2	1	2	1	8
++	0	3	2	8	1	2
+++	1	1	0	5	1	0

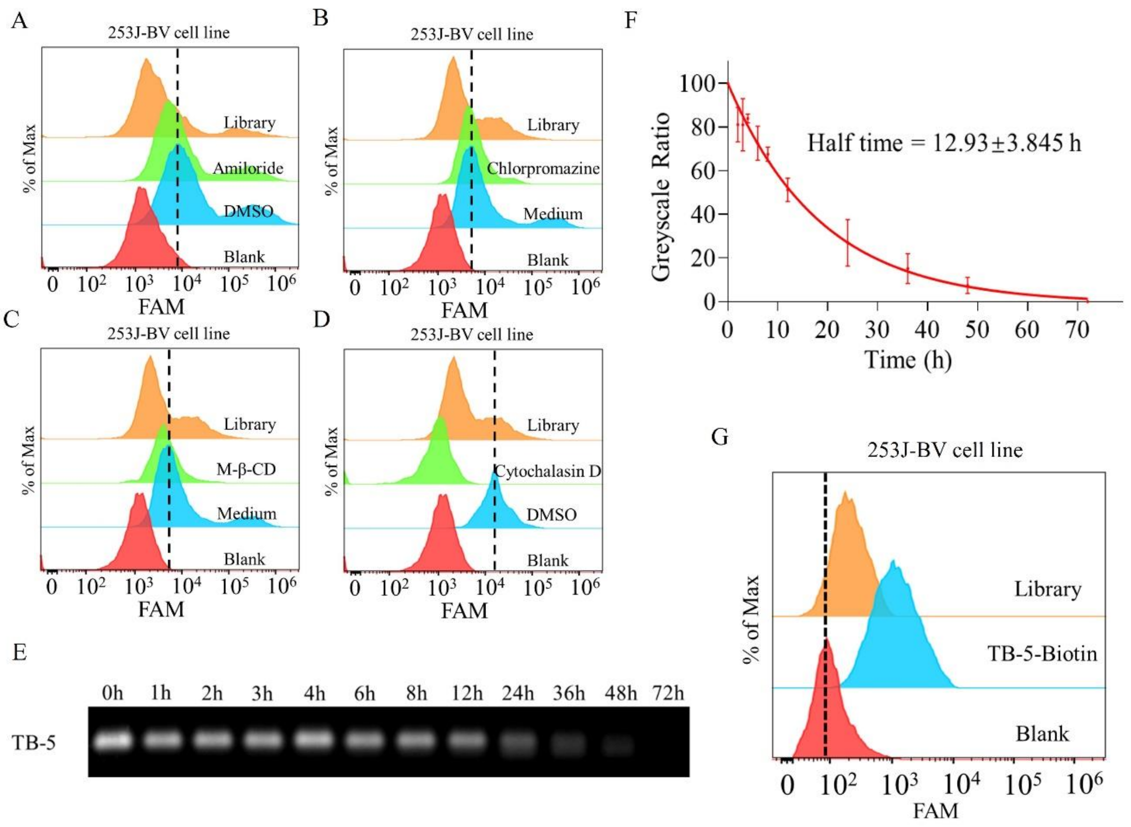

Figure S3. Endocytosis pathway analysis of TB-5 in 253J-BV cells. 253J-BV cells were separately treated with DMSO, (A) amiloride, (B) chlorpromazine, (C) M-β-CD or (D) cytochalasin D. Fluorescent signals from the uptake level of TB-5 by 5637 cells were analyzed by flow cytometry. (E) The stability of aptamer TB-5 in blood serum was determined by agarose gel electrophoresis. (F) The degradation curve and half-life of TB-5 stability. (G) The binding of biotin- and FITC-labeled TB-5 to 253J-BV cells was measured by flow cytometry, with biotin- and FITC-labeled Library as a control.

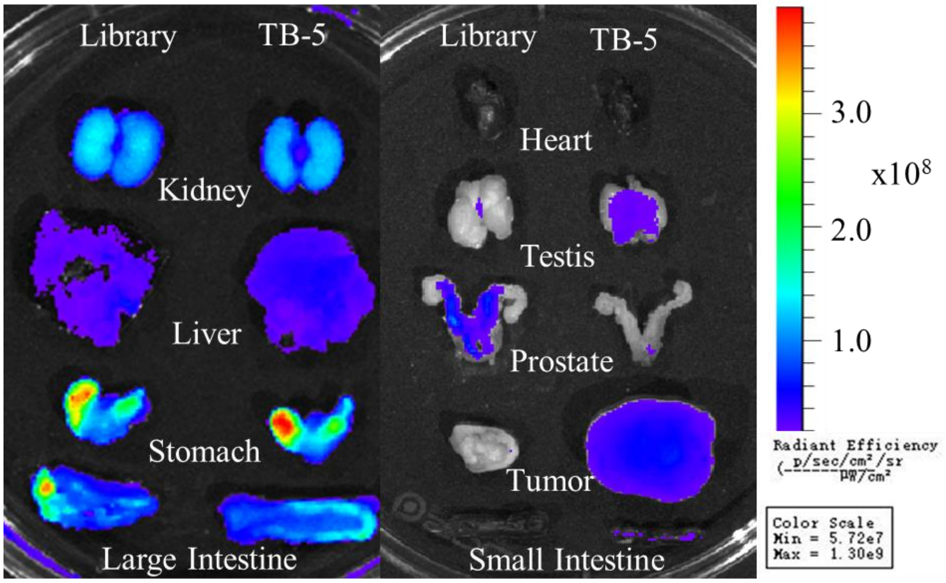

Figure S4. Fluorescence imaging of TB-5 in the organs of xenografted mouse models. Optical and fluorescent images of organs were dissected from bladder cancer (BC) -bearing mice injected with Cy5-labeled Library (left) or Cy5-labeled TB-5 (right) at 2 h post-injection.

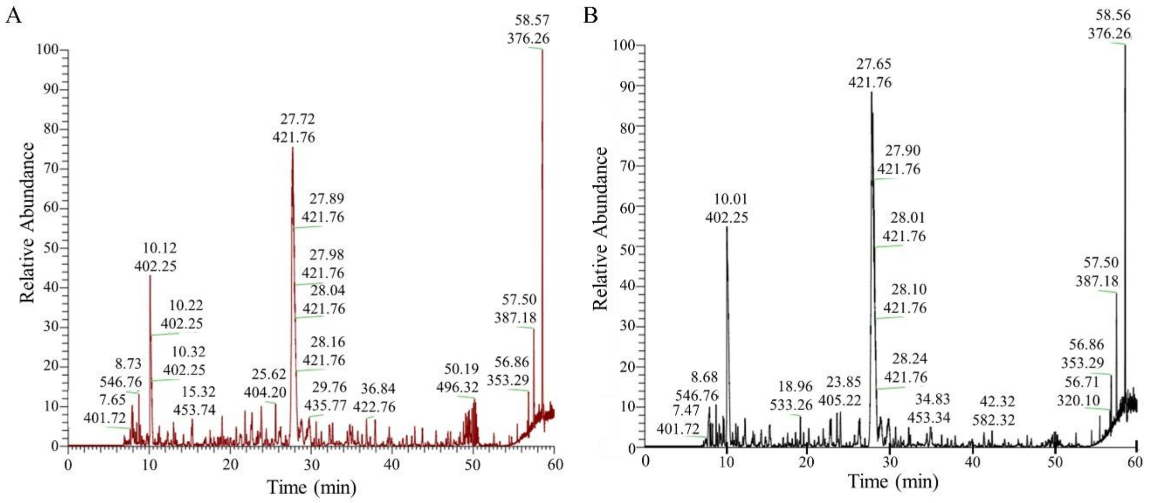

Figure S5. Representative data from liquid chromatography tandem mass spectrometry (LC-MS/MS) of a tryptic peptide from extracted membrane protein samples displayed in (A) Library (B) TB-5.

Table S7. The candidate proteins of TB-5 through mass spectrometry analysis.

Number	Name	Peptides	Unique peptides	Sequence coverage [%]	Mol. weight [kDa]	iBAQ[a] LIB	iBAQ TB-5	Ratio[b]
A0A7I2V428	Nucleolin	24	24	26.7	74.739	37384	250630000	6704.205
Q0VAS5	Histone H4	6	6	57.3	11.314	1087200	88464000	81.368653
H6VRG3	Cytokeratin-1	32	1	43.3	66.11	60454000	75738000	1.2528203
H2B1C	Histone H2B type 1-C/E/F/G/I	3	3	27.8	13.906	486040	44614000	91.790799
L0R6M0	Alternative protein PCDHGB3	1	1	15.8	6.0858	33005000	38198000	1.1573398
VSIG8	Immunoglobulin domain-containing protein 8	6	6	15.9	43.89	0	31841000	#DIV/0!
S10A3	Protein S100-A3	4	4	37.6	11.713	0	29655000	#DIV/0!
DCD	Dermcidin	2	2	17.3	11.284	43165000	28413000	0.6582416
Q4VB24	Histone cluster 1	4	3	13.7	21.893	1918400	20252000	10.556714
KR103	Keratin-associated protein 10-3	2	1	9.5	22.348	0	20085000	#DIV/0!
V9GZN0	Uncharacterized protein (Fragment)	2	2	34	5.0127	1342000	15563000	11.59687
Q3LI55	Keratin associated protein	2	2	16	17.057	0	15503000	#DIV/0!
1433S	14-3-3 protein sigma	7	4	25.8	27.774	146550	13819000	94.295462
KR161	Keratin-associated protein 16-1	6	6	11.4	53.912	0	12464000	#DIV/0!
A0A0U1RRC4	Endothelial protein C receptor (Fragment)	1	1	23.5	4.1976	0	12217000	#DIV/0!

S10A7	Protein S100-A7	2	2	23.8	11.471	22276000	11829000	0.5310199
FABP5	Fatty acid-binding protein 5	4	4	22.2	15.164	2244600	9310800	4.1480887
H9ZYJ2	Thioredoxin	2	2	21	11.737	24450000	9099200	0.3721554
KR132	Keratin-associated protein 13-2	3	3	16.6	18.727	0	7837800	#DIV/0!
E7EUT5	Glyceraldehyde-3-phosphate dehydrogenase	6	6	26.5	27.87	3142100	7524300	2.3946724
A0A5C2GJM3	IGH + IGL c207_light_IGLV1-40_IGLJ2	1	1	9.9	11.697	3675200	7257900	1.9748313
A0A804GS07	Actin, cytoplasmic 2	6	6	20.8	40.904	1717500	7100400	4.1341485
KRA47	Keratin-associated protein 4-7	4	1	18.6	22.535	0	6620800	#DIV/0!
E9PQD5	Vacuolar protein sorting-associated protein 51	1	1	8.6	17.485	4616600	6292300	1.3629728
C9J3T3	Cohesin subunit SA-3 (Fragment)	1	1	9	20.621	3252000	6202800	1.9073801
A0A0S2Z487	Junction plakoglobin (Fragment)	13	8	21.9	81.744	1389200	6190100	4.4558739
B4DVW9	cDNA FLJ51341	1	1	23.1	16.449	4603100	6088900	1.3227825
B7Z1V7	cDNA FLJ51811	2	2	3.9	47.362	6921600	5750200	0.8307617
F8VV32	1,4-beta-N-acetylmuramidase C	2	2	20.2	11.488	4545300	5476600	1.204893

a, iBAQ: intensity-based absolute protein quantification.

b, Ratio: iBAQ TB-5/iBAQ LIB; LIB : Library.

A

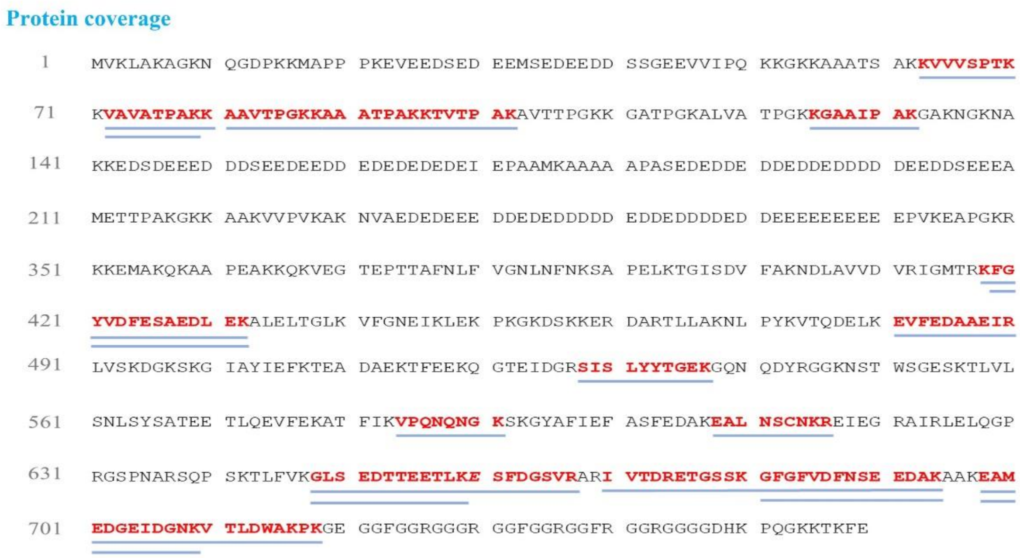

B

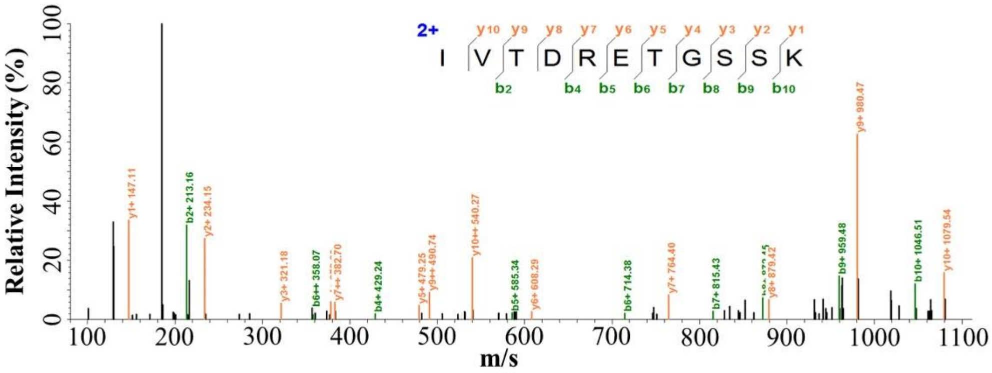

Figure S6. Protein coverage and peptide fingerprint of nucleolin (NCL). (A) Protein coverage and (B) peptide fingerprint of NCL in LC-MS/MS analysis.

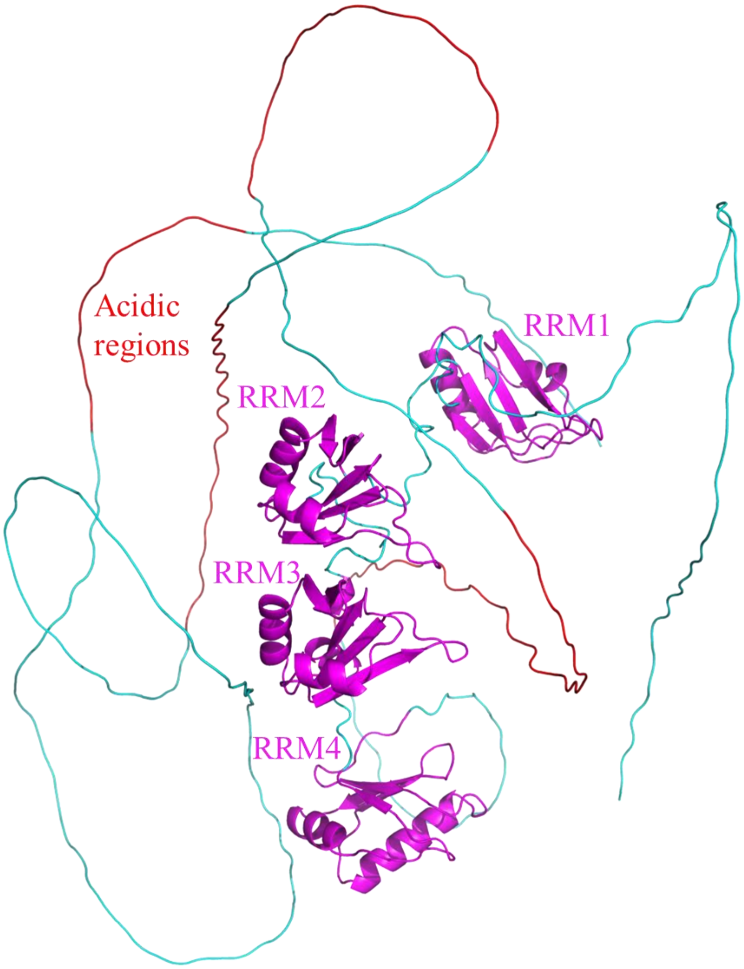

Figure S7. Schematic representation of the full-length NCL model *via* PyMOL software. The purple area shows the RNA-recognition motifs (RRMs) (RRM1:300–381; RRM2:387–470; RRM3:487–557; RRM4: 573–711;), the red area indicates the acidic regions, and the other areas are the GAR region.

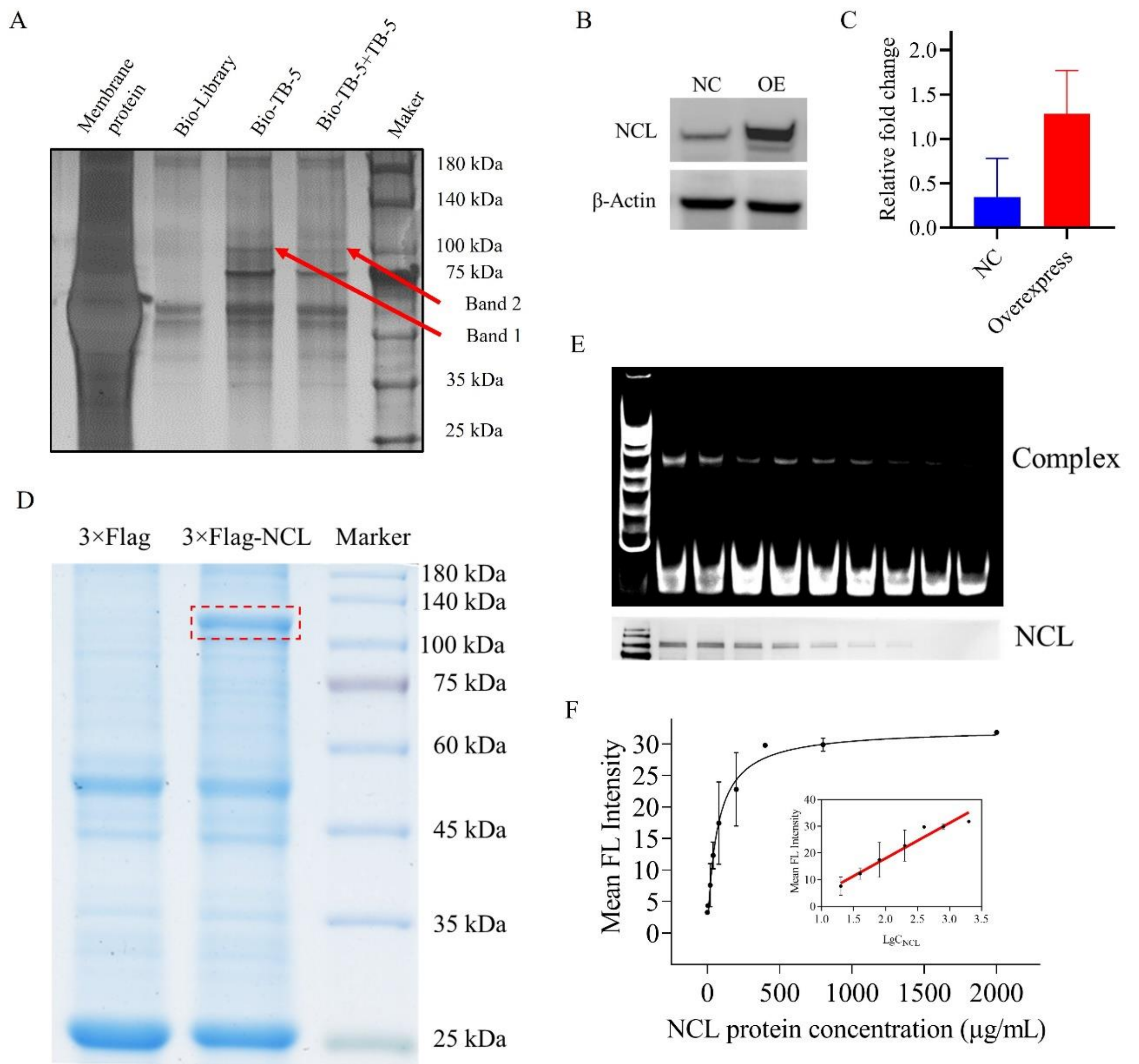

Figure S8. (A) Silver staining was used to analyze the proteins by SDS-PAGE, including total protein (Total protein), biotin-labeled-Library pulldown protein (bio-Library), biotin-labeled-TB-5 pulldown protein (bio-TB-5), and biotin-labeled-TB-5 pulldown protein after adding unlabeled TB-5 as a competitor (TB-5 + bio-TB-5). Compared with bind 1, the red arrow indicates that bind 2 is the diminished band. (B) Overexpression of NCL in HEK293 cells by Western blot. (C) Quantification of (B). (D) Coomassie brilliant blue staining of 3×Flag or 3×Flag-NCL protein expressed by the eukaryotic expression system. (E) Electrophoretic mobility shift assay (EMSA) of the binding of TB-5 to NCL *in vitro*. (F) Dissociation constant of TB-5 for 3×Flag-NCL or 3×Flag protein by EMSA.

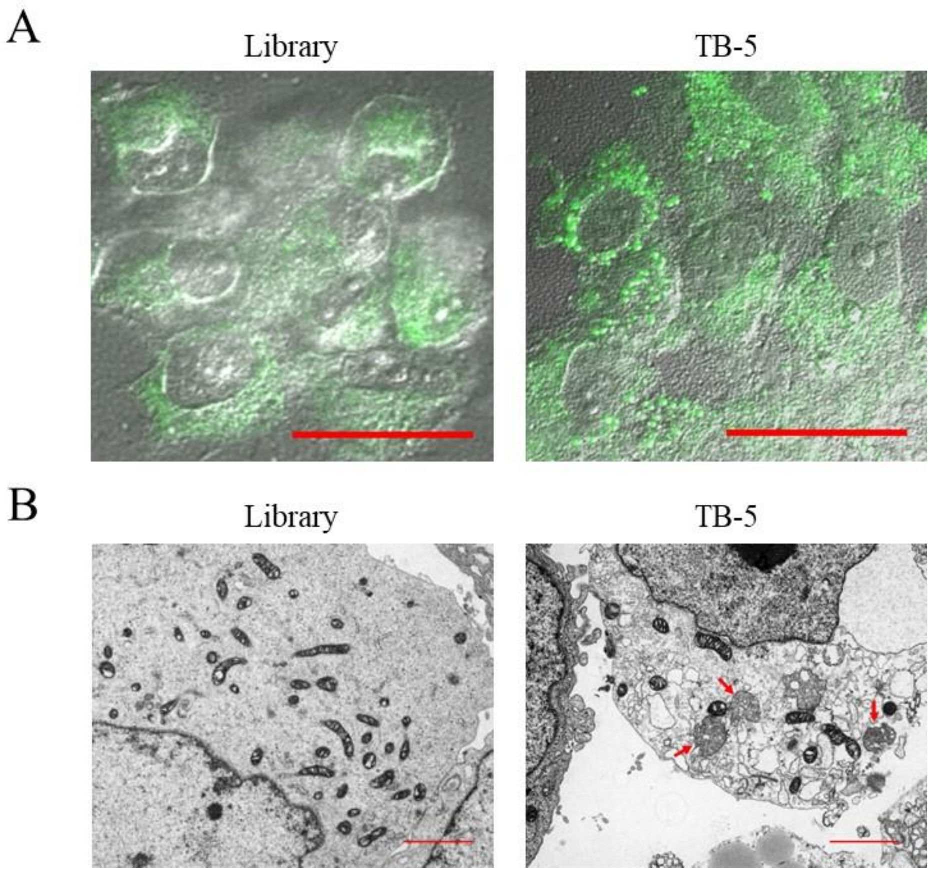

Figure S9. (A) Merged bright field images and EGFP-labeled LC3-II staining (green) (scale bar = 40 μm). (B) Representative TEM images of mitochondrial morphology in 253J-BV cells after treatment with Library and TB-5 for 72 h (Scale bar = 2 μm).

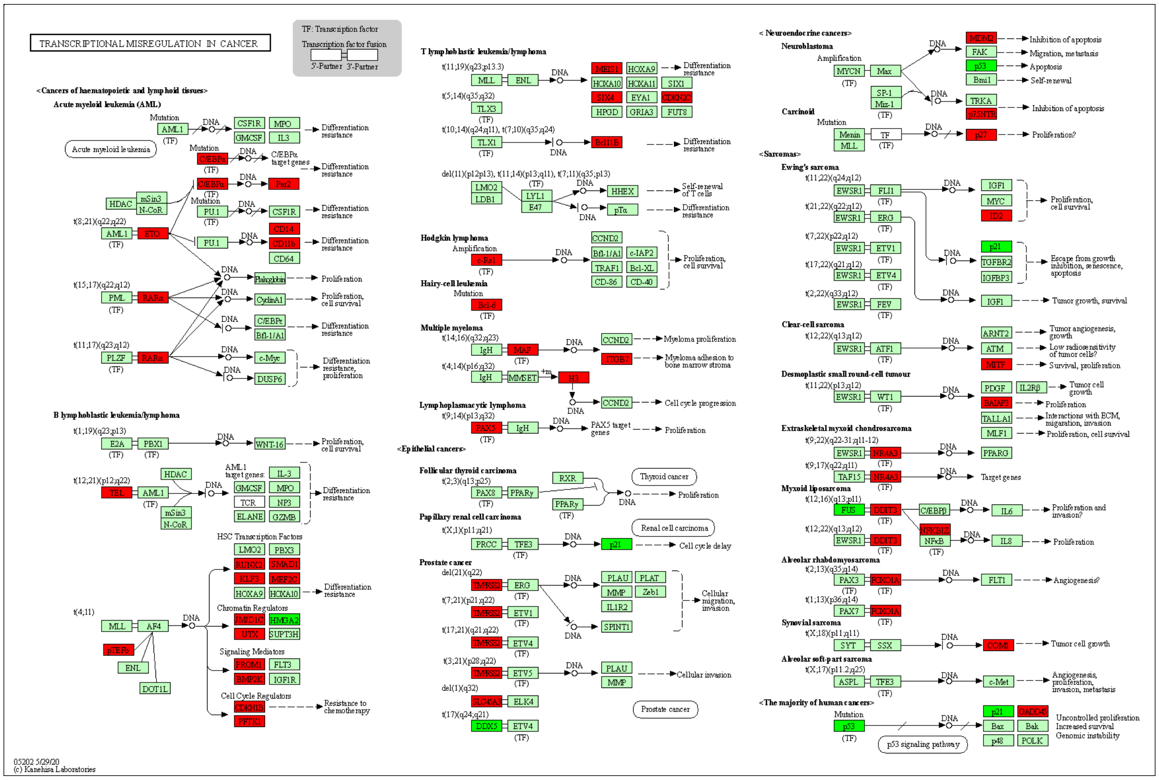

Figure S10. Kyoto encyclopedia of genes and genomes (KEGG) pathway annotation diagram of transcriptional mis-regulation in cancer.

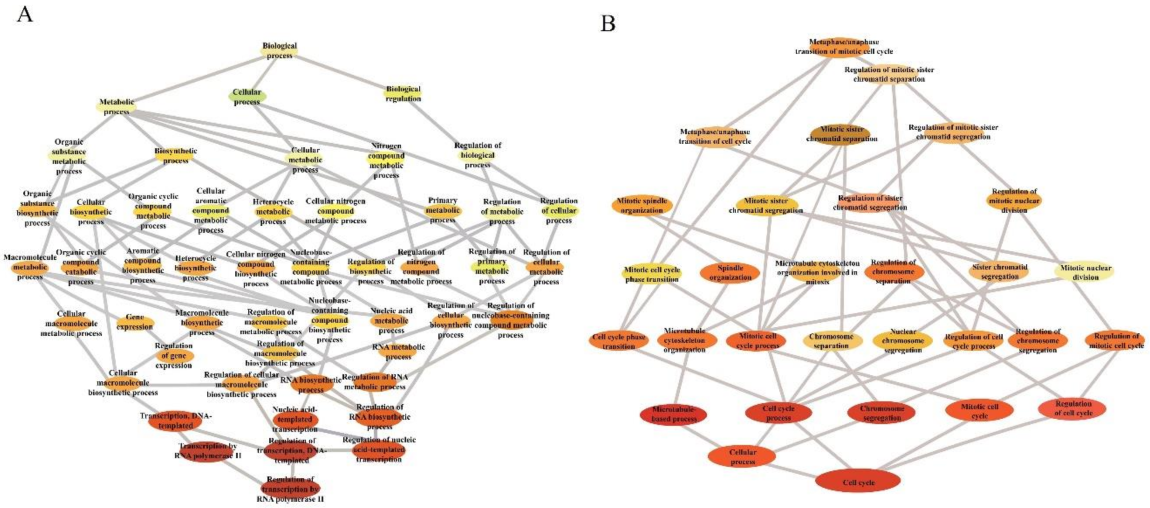

Figure S11. Gene ontology (GO) enrichment map of genes with aptamer TB-5 versus Library (A) or AS1411 versus Library (B).
